# Rhinoceros beetle horn development reveals deep parallels with dung beetles

**DOI:** 10.1371/journal.pgen.1007651

**Published:** 2018-10-04

**Authors:** Takahiro Ohde, Shinichi Morita, Shuji Shigenobu, Junko Morita, Takeshi Mizutani, Hiroki Gotoh, Robert A. Zinna, Moe Nakata, Yuta Ito, Kenshi Wada, Yasuhiro Kitano, Karen Yuzaki, Kouhei Toga, Mutsuki Mase, Koji Kadota, Jema Rushe, Laura Corley Lavine, Douglas J. Emlen, Teruyuki Niimi

**Affiliations:** 1 Division of Evolutionary Developmental Biology, National Institute for Basic Biology, Okazaki, Japan; 2 Department of Basic Biology, School of Life Science, SOKENDAI (The Graduate University for Advanced Studies), Okazaki, Japan; 3 NIBB Core Research Facilities, National Institute for Basic Biology, Okazaki, Japan; 4 Graduate School of Bioagricultural Sciences, Nagoya University, Nagoya, Japan; 5 Department of Entomology, Washington State University, Pullman, Washington, United states of America; 6 Graduate School of Agricultural and Life Sciences, The University of Tokyo, Tokyo, Japan; 7 Division of Biological Sciences, The University of Montana, Missoula, Montana, United states of America; Ludwig-Maximilians-Universitat Munchen, GERMANY

## Abstract

Beetle horns are attractive models for studying the evolution of novel traits, as they display diverse shapes, sizes, and numbers among closely related species within the family Scarabaeidae. Horns radiated prolifically and independently in two distant subfamilies of scarabs, the dung beetles (Scarabaeinae), and the rhinoceros beetles (Dynastinae). However, current knowledge of the mechanisms underlying horn diversification remains limited to a single genus of dung beetles, *Onthophagus*. Here we unveil 11 horn formation genes in a rhinoceros beetle, *Trypoxylus dichotomus*. These 11 genes are mostly categorized as larval head- and appendage-patterning genes that also are involved in *Onthophagus* horn formation, suggesting the same suite of genes was recruited in each lineage during horn evolution. Although our RNAi analyses reveal interesting differences in the functions of a few of these genes, the overwhelming conclusion is that both head and thoracic horns develop similarly in *Trypoxylus* and *Onthophagus*, originating in the same developmental regions and deploying similar portions of appendage patterning networks during their growth. Our findings highlight deep parallels in the development of rhinoceros and dung beetle horns, suggesting either that both horn types arose in the common ancestor of all scarabs, a surprising reconstruction of horn evolution that would mean the majority of scarab species (~35,000) actively repress horn growth, or that parallel origins of these extravagant structures resulted from repeated co-option of the same underlying developmental processes.

## Author summary

Goliath and Hercules beetles include some of the largest insects known, and the horns they wield are spectacular. These ‘rhinoceros’ beetles form a subfamily within the Scarabaeidae, a clade containing ~35,000 primarily hornless species. The other subfamily of horned scarabs, dung beetles, is distantly related and their horns are considered a separate origin and parallel radiation. We characterize horn development in a rhinoceros beetle and show that the details are surprisingly similar to the horns of dung beetles. Our results reveal exciting parallels at the level of underlying developmental mechanism. The superficial similarity of these two types of beetle horns mirrors an even deeper similarity in the pathways and genes responsible for their construction.

## Introduction

A variety of morphological novelties have arisen and diversified through the course of animal evolution. Where studied, preexisting genetic networks redeployed in new developmental contexts have often been found to underlie the origin of these novel morphological traits, helping explain how a restricted number of developmental genes produce a diversity of forms [1]. However, we still know very little about the details of redeployment—which genes are co-opted and why, and whether or how the particular genes co-opted facilitate or constrain the subsequent diversification of novel structures.

Beetle horns are remarkable examples of novel body parts that, once gained, are capable of radiating into a wide variety of forms. Beetle horns project from the head and/or prothorax as rigid cuticular outgrowths. Horns develop from discrete patches of epidermal tissue that detach locally from the cuticle of late-stage third-instar larvae and undergo a burst of proliferation to form a densely folded disc [2, 3]. As in the imaginal discs of *Drosophila melanogaster*, *Manduca sexta*, and other insects, the three-dimensional shape of the adult beetle horn forms first as an intricately patterned arrangement of folds in the epidermis, which then unfurls as the animal molts from a larva to a pupa [4]. Studies of horn development have focused on the genes responsible for spatial patterning and cell proliferation within these growing horn primordia. Previous work with dung beetles in the genus *Onthophagus* has shown that proximodistal patterning genes used in conventional ventral appendage development, such as antennae and legs, are important during horn development [5]. In addition, embryonic head patterning genes also likely contribute to horn formation in *Onthophagus* horns [6].

Together, these studies provide compelling evidence that redeployment of preexisting patterning gene networks underlies the evolutionary origin of beetle horns. However, studies to date have been confined almost entirely to a single genus of scarab beetles, *Onthophagus*. In fact, horns have arisen multiple times within the Scarabaeidae, and are today widespread and diverse within two divergent subfamilies of scarabs, the dung beetles (which includes *Onthophagus*), and the rhinoceros beetles (Dynastinae). Dung and rhinoceros beetles are distant clades within the otherwise-largely-hornless scarab beetles, separated from each other by approximately 150 million years [7]. Within each clade, horns appear to have arisen and been lost multiple times at different locations on the beetle (e.g., head, thorax) [8]. For this reason, dung and rhinoceros beetle horns are considered to be independent and parallel radiations of similar novel structures. Understanding whether the same or different genes underlie horn development in rhinoceros beetles, and how these genes function to form the horn compared to what occurs in dung beetles, promises critical insights to the process of modularity in evolution through gene network co-option, as well as the repeatability of evolution as it unfolds in parallel origins of elaborate and extravagant novel forms.

In the Japanese rhinoceros beetle *Trypoxylus dichotomus*, males develop a large “pitchfork” shaped horn extending from the dorsal surface of the head, as well as a shorter, curved and bifurcated horn that projects anteriorly from the prothorax. We investigated the developmental patterning and growth of *T*. *dichotomus* head and thoracic horns by performing a comprehensive search for transcription factors (TFs) and signaling molecules involved in horn formation, harnessing the power of RNA-seq. Subsequent RNAi-based functional evaluation identified 11 TFs (including developmental limb patterning genes, and head patterning genes) that contribute to horn formation in *T*. *dichotomus*, and revealed important similarities and differences in gene function between dung (*Onthophagus*) and rhinoceros (*Trypoxylus*) beetle horns. Our results point to a deep parallelism in the origin and subsequent diversification of scarab beetle horns.

## Results

### Horn primordial tissue shows sex-specific development in prepupae

**Fig 1:**
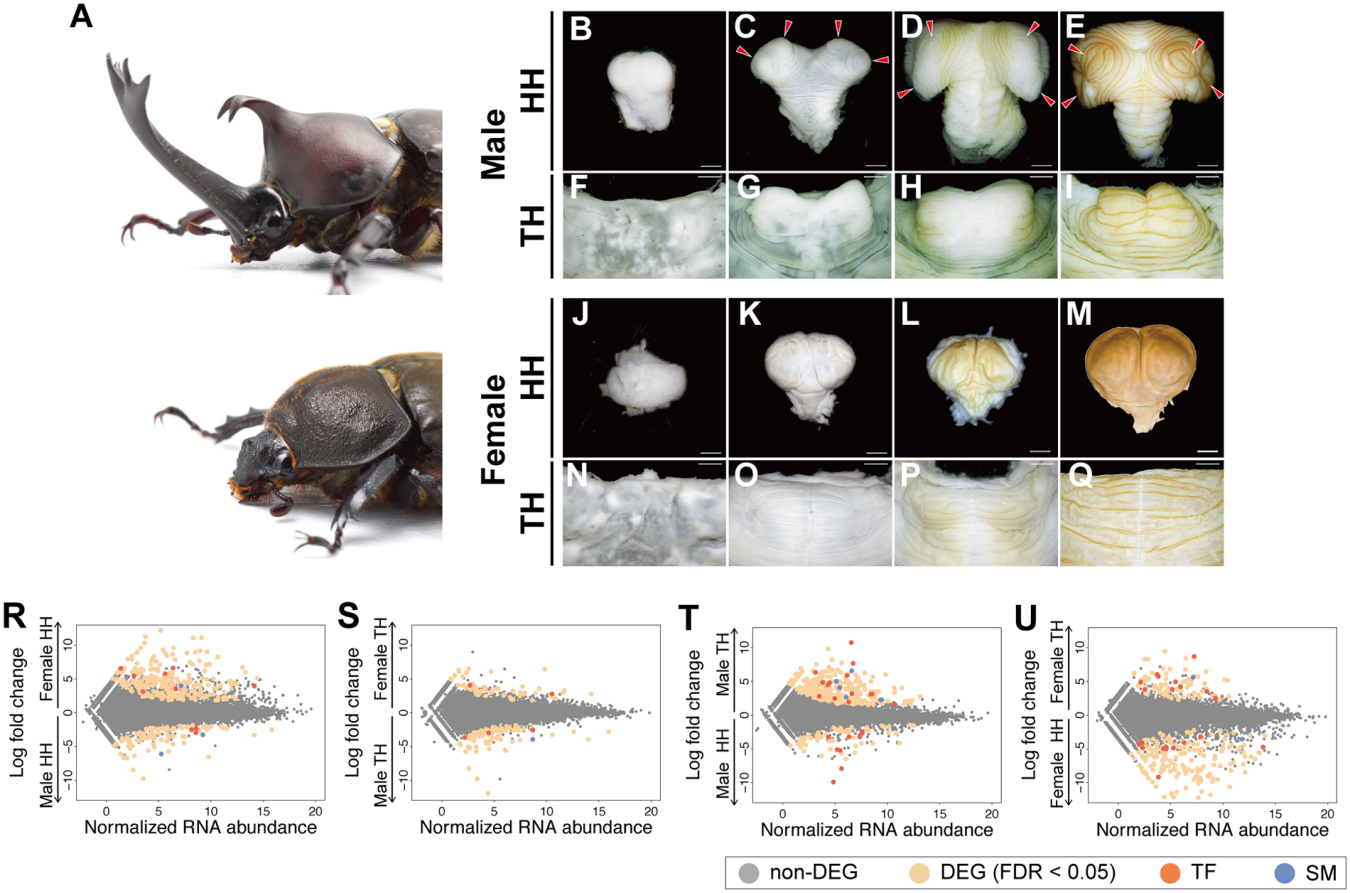
Transcription factors and signaling molecules are differentially expressed between male and female horns, and between horn types in *T*. *dichotomus*. **(A)** Male (top) and female (bottom) adult of *T*. *dichotomus*. **(B–I)** Male head (**B**–**E**) and prothoracic (**F**–**I**) horn primordia during the prepupal stage. The dorsal aspect of the horn primordium is shown. Arrowheads indicate concentric circles corresponding to the distal tips of the adult head horn. (**J**–**Q)** Horn counterparts in the female head (**J**–**M**) and prothorax (**N**–**Q**). Scale bar is 1 mm. **(R**–**U)** MA-plots for each RNA-seq data set comparison. Panels R and S show the intersexual comparisons, whereas T and U show intrasexual comparisons. Male head horn versus female head horn (**R**), male thoracic horn versus female thoracic horn (**S**), male head horn versus male thoracic horn (**T**), and female head horn versus female thoracic horn (**U**). Grey circles indicate transcripts, tan circles represent genes DEG at an FDR of < 0.05, orange circles represent TF, and blue circles represent SM. HH, head horn; TH, thoracic horn; DEG, differentially expressed gene; FDR, false discovery rate; TF, transcription factor; SM, signaling molecule.

In *T*. *dichotomus*, males develop exaggerated horns on both the head and thorax while female beetles are hornless (Fig 1A). In order to determine when male-specific horn morphogenesis begins, we first compared the development of male horn primordia and tissue from the same region in females. In *T*. *dichotomus*, sexually dimorphic horn development becomes apparent during the prepupal stage [9]. Approximately ten days prior to the end of the last (third) larval instar of males, cells of the dorsal head epidermis begin to evaginate to form a sac (Fig 1B). The surface of the sac continues to grow and fold during prepupal development, and forms four concentric circles at its distal tips that correspond to the branched tips of the adult head horn [3] (arrowheads in Fig 1C–1E). The development of the thoracic horn follows a similar progression, although the onset of evagination occurs later than in the head horn (Fig 1F–1I). In addition, the thoracic horn forms a different surface folding pattern than head horns, reflecting the differences in adult horn shape.

In *T*. *dichotomus*, the female pupa has a small protrusion on the head, and no visible horn on the thorax. This small pupal head horn in females disappears after eclosion, likely through programmed cell death during the adult molt [10]. The primordial tissue for the head structure in prepupal females displays less folding and lacks the four concentric circles typical of male horns (Fig 1J–1M). Prepupal female tissue located at the same region as male thoracic horn tissue, while still displaying a folded morphology, displays less folding than male tissue and lacks the morphology of male thoracic primordia (Fig 1N–1Q). Consistent with their different morphology at the pupal stage, females show no clear evagination in their horn primordia late in development (Fig 1M and 1Q).

To discover horn formation genes we sampled horn primordia at the onset of differentiation (Fig 1C, 1G, 1K and 1O) because (i) male-specific tissue folding is present during this stage, and (ii) the primordia are more clearly recognizable than in earlier stages, enabling consistent tissue collection among samples.

### Discovering 11 transcription factors that affect horn formation in *T*. *dichotomus*

We then performed RNA-seq analysis to identify genes that contribute to elaborate horn morphology. To construct a *T*. *dichotomus* transcriptome for read mapping, we assembled four cDNA libraries, comprising male head horn tissue (111.2M reads, see Materials and methods for detail), male thoracic horn tissue (107.8 M reads), female head horn tissue (113.6 M reads) and female thoracic horn tissue (115.1 M reads). The summary of sequencing and *de novo* transcript assembly is shown in S1 Table. The total number of trinity transcripts is 82,108 and the contig N50 based on all transcript contigs is 3,158 (S1 Table). We evaluated the quality of the assembled transcript model by performing a BLAST search against both the NCBI nr database as well as the OrthoDB 5 database. The assembled transcripts showed the highest similarities to Coleopteran (beetle) genes, particularly to genes of the red flour beetle *Tribolium castaneum* in the NCBI nr database search (S1A–S1C Fig). In the OrthoDB search, 75.3% of the assembled transcripts had putatively orthologous genes in *D*. *melanogaster* and 89.3% of the transcripts were orthologous to *T*. *castaneum* (S1D and S1E Fig). As *T*. *dichotomus* and *T*. *castaneum* are both beetles, the increased percentage of transcripts orthologous to *T*. *castaneum* demonstrates the quality of the *T*. *dichotomus* transcriptome constructed in this study. We further evaluated our transcriptome with BUSCO [11]. Our transcriptome indicates 97.8 and 95.7% coverages over complete BUSCOs in Metazoa and Insecta, respectively (S1F Fig), reflecting the high quality of the *T*. *dichotomus* transcriptome.

We mapped short read sequences to the transcriptome and calculated mRNA abundance. We checked the distribution of count data with a multi-dimentional scaling plot, and found that gene expression between males and females was distributed distinctly, and that at least two biological replicates for each sample clustered together (S2 Fig). We then made two different types of comparisons to identify transcripts involved in horn formation. First, we made an intersexual comparison between the same horn types of male and female beetles (e.g. between head horns in male and female). This was performed in order to identify genes driving the development of different morphologies between male and female horns. Next, we compared tissue intrasexually, between different horn types in either males or females (e.g. between head horn and thoracic horn in males). Our goal was to identify genes that contribute to the unique horn morphologies present in each segment. Our intersexual comparison identified 739 differentially expressed genes (DEGs), and our intrasexual comparison identified 814 DEGs at a false discovery rate lower than 0.05 (S2 Table; S1–S4 Appendices).

To understand the developmental processes each tissue undergoes during the stage we investigated, we categorized genes enriched in each comparison by gene set analysis using ErmineJ software [12]. In our intersexual comparisons, Gene Ontology (GO) terms associated with muscle formation (e.g. myofibril assembly, striated muscle cell development) and metabolism (e.g. cellular amino acid catabolic process, lauric acid metabolic process) were overrepresented (S3 Table; S3 Fig). This suggests that sex differences in horn morphology (male versus female head horn, male versus female thorax horn), at least during the prepupal period (Fig 1C, 1G, 1K and 1O), arise primarily from differences in the amount of growth of each horn type. In contrast, differences between horn types (head horn versus thorax horn) are associated with differential expression of appendage patterning genes. GO terms associated with morphological differentiation, such as cell fate specification, leg disc pattern formation and head development, were clearly enriched in the comparisons between head and thoracic horns within each sex (S3 Table; S3 Fig).

### RNAi-mediated knockdown affects horn formation: *SP8* and *pnr*

We evaluated the function of DEGs during horn formation by using RNAi-mediated gene knockdown. Given the important functions of transcriptional regulation in animal development, we focused on DEGs annotated either as transcription factors (TFs) or as signaling molecules for our RNAi screening. In our annotation based on BLAST search against the NCBI nr database, we identified 49 candidate horn formation genes comprising 38 TFs and 11 signaling molecules (Fig 1R–1U; S4 Table). We first performed RNAi for all 49 candidate horn formation genes as an initial screening, and repeated experiments for 13 TFs and a signaling molecule in which we observed visually detectable effects on head and/or thoracic horn morphology (S4 Table). We consequently obtained 11 TFs with clear functional roles in horn development (S4 Table).

**Fig 2:**
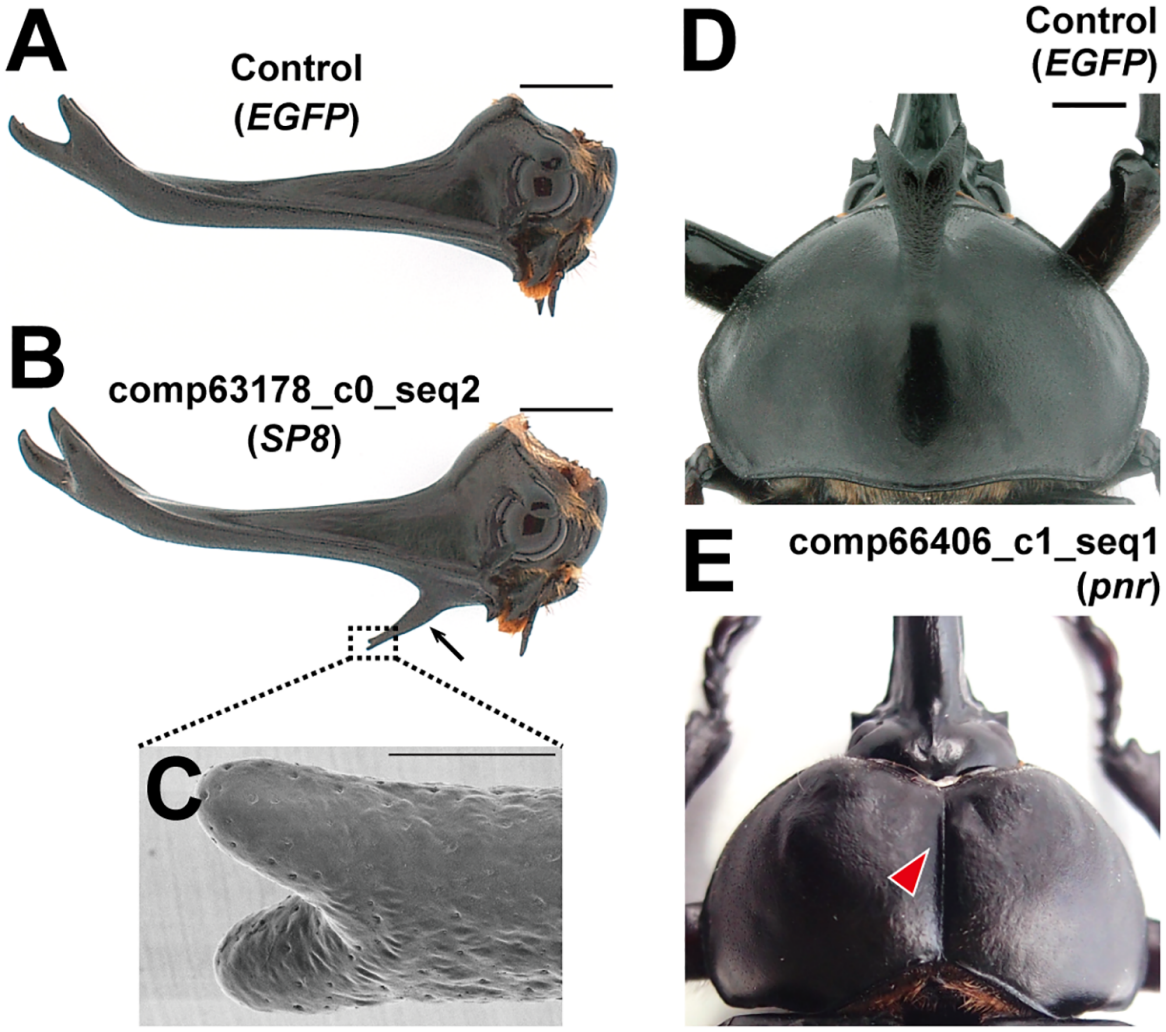
Extreme horn phenotypes after *SP8* and *pnr* RNAi. **(A**, **B)** Lateral view of adult heads from *EGFP* (**A**) and *SP8* (**B**) RNAi beetles. The arrow in **B** indicates the ectopic horn. (**C)** A magnified SEM image of an ectopic horn in a *SP8* RNAi beetle from ventral view. (**D**, **E)** Dorsal view of adult prothorax areas of *EGFP* (**D**) and *pnr* (**E**) RNAi beetles. Arrowhead indicates the furrow formed along dorsal midline instead of a thoracic horn. Scale bars are 5 mm in **A** and **B**, 1 mm in **C**, 3 mm in **D** and **E**.

**Fig 3:**
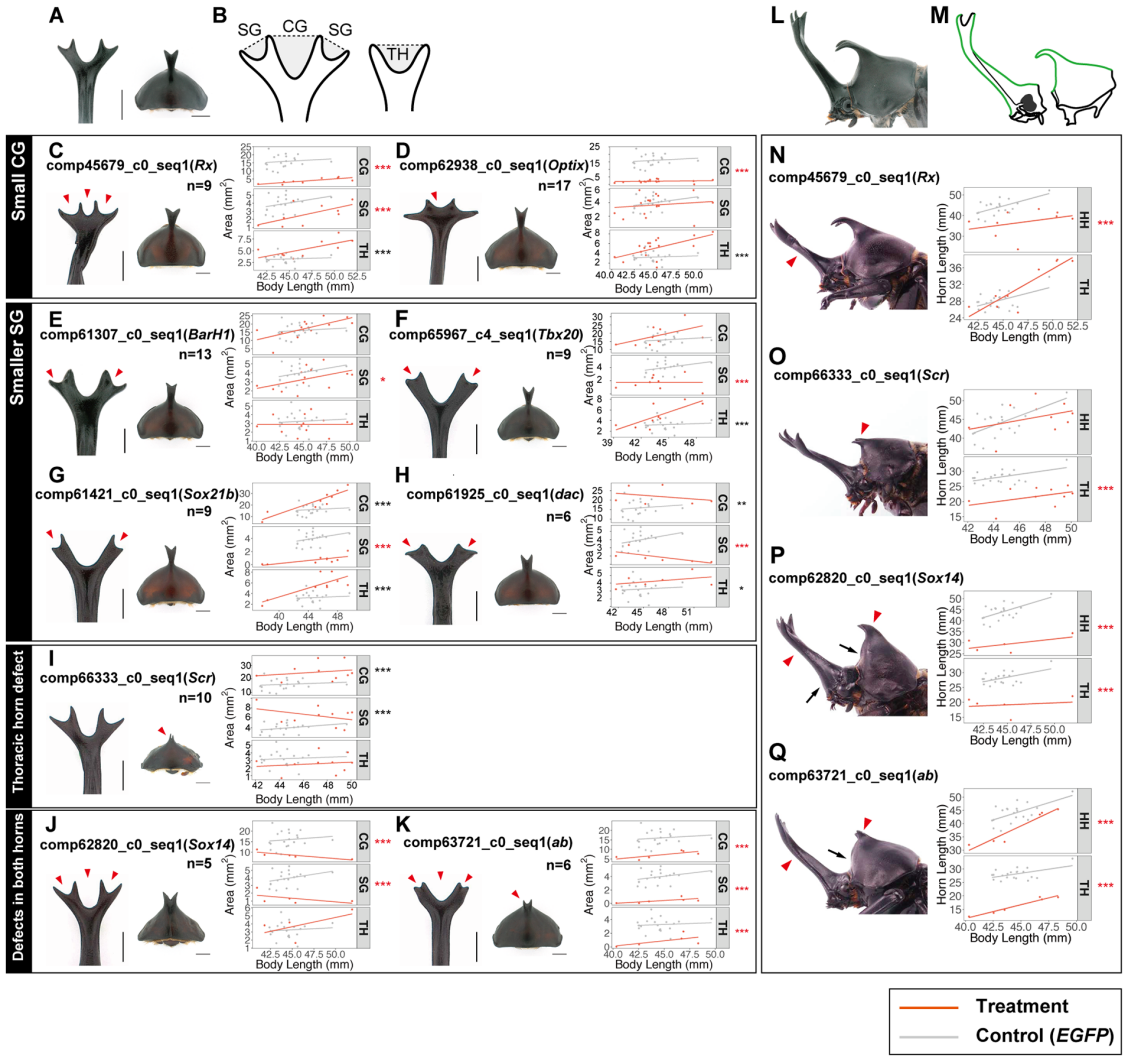
RNAi-mediated gene knockdown alters horn shapes and sizes in head and thorax. **(A**–**K)** RNAi effects on horn shapes. (**A)** Dorsal view of head horn and prothorax in an *EGFP* RNAi beetle. (**B)** Schematics indicate measured regions. Gray shaded regions are a central groove (CG) and side grooves (SGs) in head horn, and a groove in thoracic horn (right). (**C**–**K)** Dorsal view of head horn and prothorax in RNAi beetles are displayed. Arrowheads indicate significantly decreased areas. Horn area and body length are plotted, and linear regression lines are drawn for each gene in red. Gray dots and gray regression lines are for *EGFP* RNAi beetles. Asterisks show significance of each RNAi treatment using a Wald test on the logistic regression. Asterisks for significantly affected areas are in red. Asterisks denote significance: * P < 0.05, **P < 0.01, ***P < 0.005. Scale bars are 5 mm. **(L**–**Q)** RNAi effects on horn size. (**L)** Lateral view of head and prothorax in an *EGFP* RNAi beetle. (**M)** Green lines indicate measured segments. (**N**–**Q)** Lateral view of head and prothorax in RNAi beetles are displayed. Horns that are statistically shorter than controls are denoted with arrowheads, whereas arrows indicated abnormally wider proximal parts in *Sox14* (**P**) and *ab* (**Q**) RNAi beetles. Horn length and body length are plotted, and linear regression lines are drawn for each gene in red. Gray dots and gray regression lines are for *EGFP* RNAi beetles. P-values show significance of each RNAi treatment according using Wald test. Asterisks denote significance: * P < 0.05, **P < 0.01, ***P < 0.005.

Among the 11 genes we identified, *SP8* and *pannier* (*pnr*) RNAi most drastically affected horn phenotypes. SP8 is a member of the SP family of transcription factors, and has an orthologous amino acid sequence to *D*. *melanogaster* Sp1 (S4 Fig). In *D*. *melanogaster*, SP family genes *Sp1* and *buttonhead* (*btd*) play partially redundant roles in development of ventral appendages and mechanosensory organs [13–15]. *D*. *melanogaster* Sp1 is involved in leg disc fate determination and postembryonic growth of ventral appendages [13, 14]. The role of a *D*. *melanogaster* Sp1 ortholog on growth of ventral appendages appears to be conserved in the beetle *T*. *castaneum* [16]. There are three sets of SP family genes in metazoans [17], and while members of all three families were present in our transcriptome, *SP8* was the only SP gene identified as differentially expressed. *SP8* knockdown induced an extra horn-like outgrowth from the anterior proximal region of the male head horn (Fig 2A and 2B). Head horns in *T*. *dichotomus* are unusual in having four tips, suggesting two successive bifurcation events (Fig 3A). This new RNAi-induced horn outgrowth—a horn on a horn—exhibited a bifurcated tip (Fig 2C), similar to the bifurcated tip of *T*. *dichotomus* thoracic horns (Fig 3A), as well as head horns of other, more typical, Dynastinae species.

In *D*. *melanogaster pnr* expression is localized to the dorsal midline, where it acts as a selector gene specifying dorsomedial identity within the head and thorax [18]. *pnr* also specifies dorsal regions of eye-antennal and wing imaginal discs and is an upstream regulator of both *decapentaplegic* (*dpp*) and *wingless* (*wg*), making it an ideal candidate for a master regulator of patterning and growth of head and thoracic horns [19]. Knockdown of *pnr* led to the development of a furrow along the dorsal midline of the T1 segment of the thorax, instead of a horn (Fig 2D and 2E). This complete loss-of-horn phenotype strongly suggests that *pnr* also functions as a dorsal-medial selector in *T*. *dichotomus* prepupal development. Interestingly, *pnr* did not affect development of the head horn, which suggests that cells giving rise to head horns may not be dorsal in origin—a possibility we discuss further below.

The remaining genes analyzed in our RNAi screening displayed only modest phenotypes, and thus we quantitatively assessed the effect of RNAi treatment by measuring both horn shape and length (S4 Table).

### Nine additional transcription factors contribute to a characteristic horn shape and size

#### Horn shape

*T*. *dichotomus* head horns have a double-branched distal tip with a deep central groove and two shallower lateral grooves (Fig 3A). Thoracic horns have a small central groove at the distal tip. We measured the area of the central and lateral grooves in head horns and the central groove in thoracic horns as proxies for horn shape (Fig 3B; S5 Table; S5 Appendix). Since horn shape is prepatterned through folding of the horn primordia during the prepupal stage, any changes in horn shape likely reflect an effect of RNAi treatment on this underlying folding pattern (Fig 1B–1I).

For head horns, RNAi treatments had different effects on the central and lateral grooves (Fig 3C–3H). *Rx* and *Optix* RNAi beetles had a significantly smaller central groove area than controls (Fig 3C and 3D). On the other hand, RNAi knockdown of *BarH1*, *dac*, *Sox21b* and *Tbx20* resulted in reduced side groove areas, but not central groove areas (Fig 3E–3H). We found no changes in shape (i.e. reduction of groove size) in thoracic horns from RNAi of these six genes (Fig 3C–3H).

The homeotic gene *Sex combs reduced* (*Scr*) is required for prothoracic character formation [20]. RNAi knockdown of *Scr* in *T*. *dichotomus* consistently affected the shape of the thorax horn, reducing the size of the thoracic horn groove, although the magnitude of this effect was not statistically significant (Fig 3I). We found defects in both head and thoracic horns in *Sox14* and *ab* RNAi beetles, and it is possible that these effects are due to the RNAi treatment affecting segment formation (Fig 3J and 3K; S5 Fig).

#### Horn size

To assess the effects of RNAi on horn size, we also measured horn length (Fig 3L and 3M; S6 Table; S6 Appendix). Although both *Rx* and *Optix* RNAi caused similar changes in central groove formation in head horns, only *Rx* RNAi decreased head horn length (Fig 3N; S6 Fig). *Optix* RNAi showed no effect on head horn length, which suggests that these genes have independent functions (S6 Fig).

In *Onthophagus* dung beetles *Scr* has a prominent role in thoracic but not head horn development, with RNAi knockdown reducing horn size in two species [21]. In addition to the function in horn shape, we also detected a significant change in thoracic but not head horn size in *Scr* RNAi beetles, consistent both with *Onthophagus* and with the known *Hox* function of *Scr* in the prothorax (Fig 3O). For *Sox14* and *ab*, we detected clear effects on the length of both head and thorax horns, providing more evidence for the function of these genes in both regions suggested by our horn shape analysis (Fig 3P and 3Q). We note that *Sox14* RNAi beetles also showed abnormal shapes of overall segments, which suggests that this gene might have broader functions in overall body patterning, rather than specific effects on horns (S5 Fig). Indeed, Sox14 is required to transduce ecdysone signal, which is a key regulator of molting and metamorphosis in insects, and thus has a broad morphogenetic role in postembryonic development [22].

### Common gene functions between female pre-segmental regions and male head horns suggest roles of clypeolabrum formation genes in horn shape, size and number

To investigate the regulatory and functional relationships among our 11 genes of interest, we searched the integrated *D*. *melanogaster* genomics database FlyMine using *D*. *melanogaster* orthologs as a query [23] (S7 Table). Although we found no pathway enrichment that suggests cooption of a specific developmental signal from this analysis, we found two terms from the Berkeley Drosophila Genome Project (BDGP) that were significantly enriched. These terms are assigned based on expression patterns of the genes in *D*. *melanogaster* embryonic development, and both the terms “clypeolabrum” (P = 2.022393e^-4^, Holm-Bonferroni test) and “clypeo-labral primordium” (P = 0.020819, Holm-Bonferroni test) were significantly enriched (S7 Table), suggesting that our 11 candidate genes are involved in formation of this region. This finding agrees with data from *Onthophagus* beetles, where it has been reported that head horns are anatomically positioned around the pre-segmental ocular and clypeolabral regions, and genes involved in the patterning of embryonic pre-segmental regions are important for post-embryonic head horn differentiation [6].

The arthropod labrum originates as an ectodermal outgrowth arising just in front of the mouth (i.e., pre-segmental), in a domain of the head defined by expression of *Optix* [24]. Whether the labrum is a true appendage, or instead a non-appendicular projection, is debated [25]; but it forms in many insects (including *T*. *castaneum* beetles) from a pair of appendage-like outgrowths that later fuse medially into a single structure [24]. In *T*. *castaneum*, appendage patterning genes including *dpp* and *wg* are expressed in labral buds, but their domains of expression are reversed compared with other trunk appendages, leading Posnien *et al*. (2009) to propose that the labrum arose as an anterior outgrowth of the head from an ectopic redeployment of the appendage patterning network [24].

**Fig 4:**
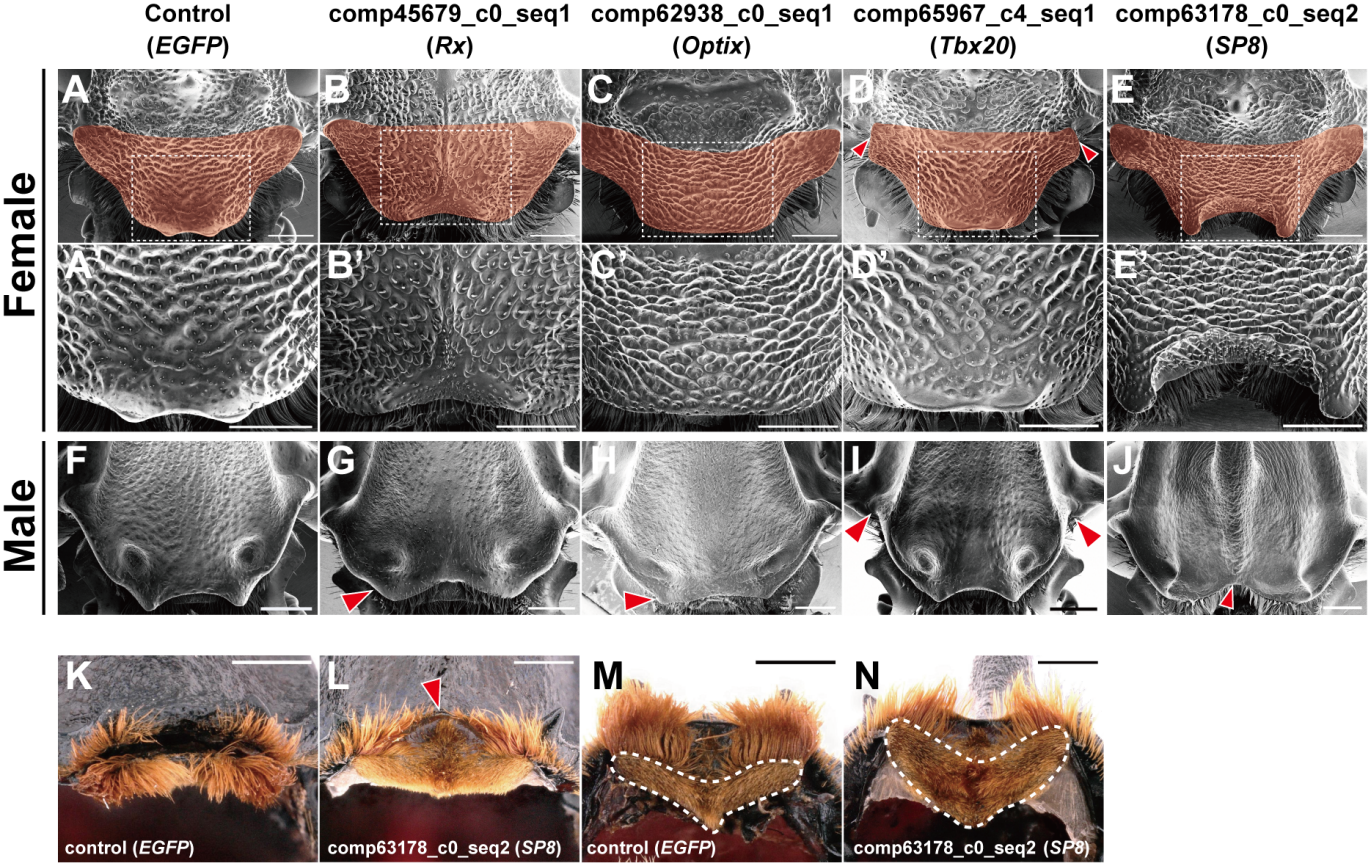
Depletion of “clypeolabral” genes affected formation of pre-segmental region in both females and males. **(A**–**E)** Anterior region of adult female heads after RNAi treatment are displayed in a dorsal view, with the clypeolabral regions in red. Arrowheads in **D** indicate defects in the lateral section of the clypeolabral region in a *Tbx20* RNAi beetle. Regions boxed with white dotted lines in **A**–**E** are magnified in **A’**–**E’**. (**F**–**J)** Corresponding head regions in male RNAi beetles. Arrowheads in **I** indicate defects at lateral parts. (**K**–**M)**, *EGFP* (**K** and **M**) and *SP8* (**L** and **N**) RNAi beetles from frontal (**K** and **L**) and ventral (**M** and **N**) views. Arrowhead in **L** indicates the loss of dorsal part of labrum in an *SP8* RNAi beetle. The ventral side of labrum is in dotted line in **M** and **N**. Scale bar is 1 mm.

Our observation that genes functionally involved with head horn growth are associated with clypeolabral identity led us to hypothesize that the novel head horn in *T*. *dichotomus* is at least partially derived from pre-segmental regions, and that some of the RNAi induced changes in horn shapes we observed in this study were a consequence of perturbing development in the clypeolabral region. To test this hypothesis, we first analyzed the phenotype of the anterior head in RNAi treated females, as the morphology of the pre-segmental region is much clearer in hornless females than in males (Fig 4A). We analyzed female heads for genes which were assigned the BDGP term “clypeolabrum” in the FlyMine analysis including *Rx*, *Optix*, *SP8* and *Tbx20* (S7 Table). *Rx* and *Optix* are required to form the larval clypeolabral region in *T*. *castaneum* [26]. *Sp1* loss-of-function mutants in *D*. *melanogaster* lack the mandibular head segment tissue (note that the *D*. *melanogaster Sp1* ortholog in beetles is *SP8*, see S4 Fig for SP family gene phylogenetic tree) [17, 27]. Although *midline*, a *Tbx20* homolog, is expressed in the clypeolabral region in *D*. *melanogaster* embryos, no head patterning function has yet been reported for any *Tbx20* homologs in insects.

In female *T*. *dichotomus*, the anterior region of the clypeus has a characteristic curved shape, and has shorter hairs on the surface than the posterior region (Fig 4A and 4A’; **clypeolabral region is in red**). *Rx* RNAi in females created a furrow in the clypeolabral dorsal midline, and dramatically changed both the shape and the surface hair pattern (Fig 4B and 4B’). *Optix* RNAi females showed a wider anterior end compared to the control counterparts (Fig 4C and 4C’). *Tbx20* RNAi resulted in a less pronounced anterior curved shape, and the posterior part of the clypeolabral plate was less extended laterally (Fig 4D and 4D’, **arrowheads**). We found that male beetles exhibited similar shape changes in the anterior head region compared to controls, indicating that these gene functions are shared between sexes, despite the corresponding body wall changes in males that ultimately form the horn (Fig 4F–4I).

We further discovered that *SP8* RNAi, which forms a small ectopic horn in males, also affected the clypeolabrum shape in both males and females. In *SP8* RNAi females, the anterior central part of the head has a u-shaped form, a rough surface, and long hair (Fig 4E and 4E’). Similar changes in anterior head shape were observed in male *SP8* RNAi beetles (Fig 4J). The labrum and clypeus are separate in control beetles, but were instead fused in *SP8* RNAi males (Fig 4K and 4L). The labrum segment lost nearly completely the normal dorsal structure, and the hairy ventral region was expanded in treated animals (Fig 4M and 4N).

These drastic changes of the clypeolabrum structure that coincide with ectopic horn formation suggest that the anterior-most tissue of the head has lost its identity, and that both dorsal and ventral characters are juxtaposed in atypical ways in *SP8* RNAi animals. Artificial juxtapositions of dorsal-ventral signals in *D*. *melanogaster* imaginal discs can lead to new axes of outgrowth and ectopic miniature wings or legs that form at the base of existing structures [28, 29]. It is conceivable that altered dorsal-ventral patterning in the *T*. *dichotomus* clypeolabral region created a new center for horn growth, and thus induced the formation of a small horn following *SP8* RNAi.

Combined, our analyses reveal that *Rx*, *Optix*, *Tbx20* and *SP8* function in the formation of both the male and female clypeolabral region and in the male head horn, and changes in the expression level of these genes alter horn shape and size.

### Appendage patterning genes play roles in *T*. *dichotomus* horn formation

**Fig 5:**
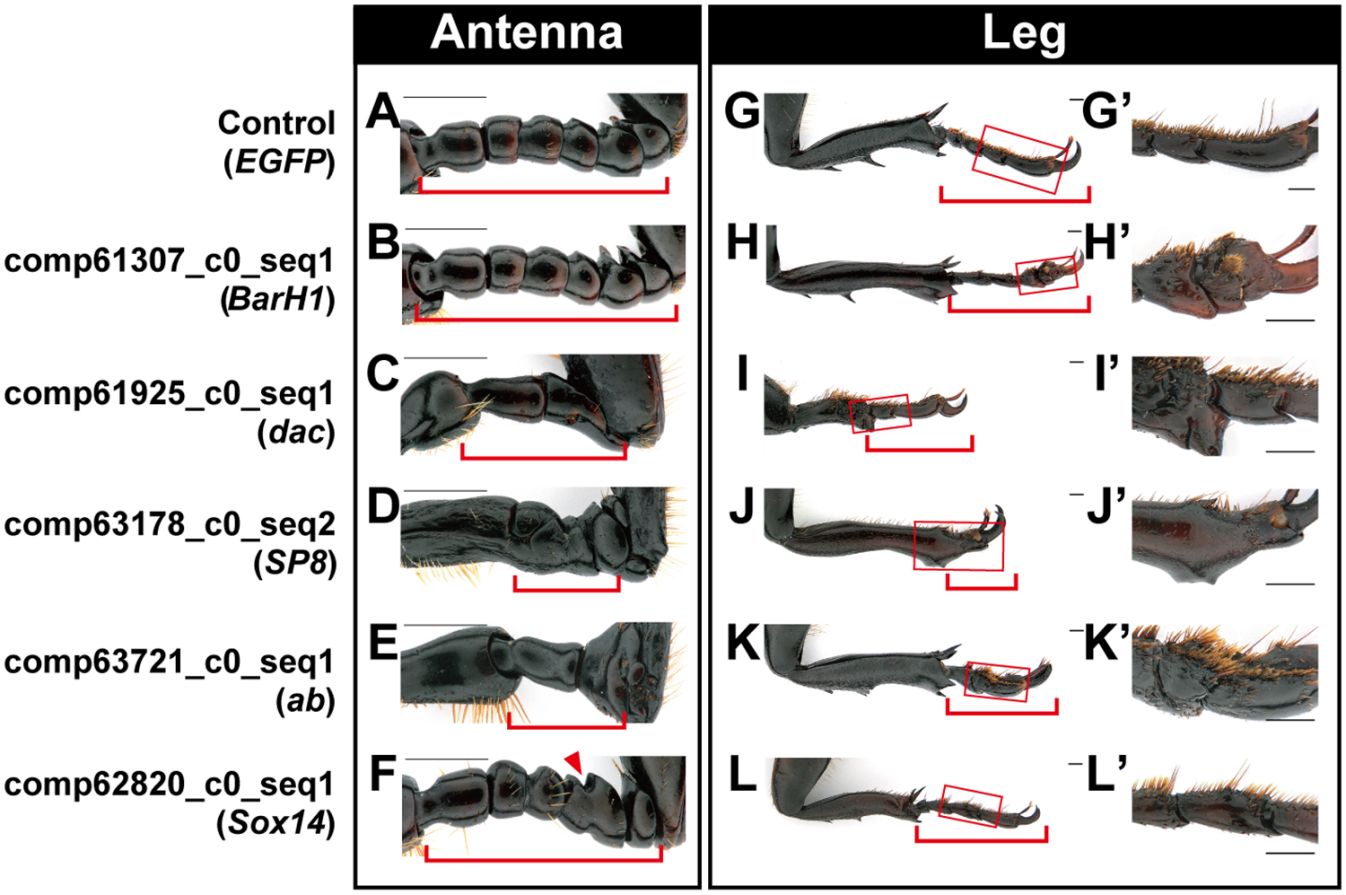
Five genes showed defects in appendage formation after RNAi. **(A**–**F)** Antennae in RNAi beetles. **(G**–**L)** Metathoracic legs in RNAi beetles. Arrowhead indicates fusion of segments. Corresponding parts are indicated with red parentheses for antennae (**A**–**F**) and legs (**G**–**L**), respectively. Boxed regions in **G**–**L** are magnified in **G’**–**L’**. Scale bar is 1 mm.

Previous work suggests that appendage patterning gene networks were coopted to form novel horn outgrowths in *Onthophagus* beetles, as both appendages and horns deploy similar developmental pathways [5]. We thus hypothesized that differentially expressed genes in our study would include appendage patterning genes. Four of our 11 genes are homologs of known appendage formation genes in *D*. *melanogaster*: *BarH1*, *dac*, *SP8* and *ab* [13, 30–32]. RNAi knockdown of these four genes led to defects in both antenna and leg development, confirming their functional role in appendage growth in *T*. *dichotomus* (Fig 5A–5E, 5G–5K and 5G’–5K’). In addition, *Sox14* RNAi led to fusion of appendage segments in the distal tip, a phenotype that has not been reported for this gene in any other insect (Fig 5F, 5L and 5L’). RNAi knockdown of *dac*, and *ab* affected both head and thoracic horn shape (Fig 3E, 3H and 3K), and *ab* affected the size of both horns (Fig 3Q). Our finding that *dac* plays a role in horn shape formation in *T*. *dichotomus* is noteworthy because RNAi knockdown of *dac* does not affect *Onthophagus taurus* horn formation [5]. While *SP8* was assigned the BDGP term “clypeolabrum”, and functions in patterning this region, *BarH1*, *dac*, *ab*, *and Sox14* were not assigned this term (S7 Table), suggesting independent recruitment of appendage patterning genes into the horn development program.

### Differential function of clypeolabral genes and conserved function of appendage genes between *T*. *castaneum* and *T*. *dichotomus*

To understand the ancestral function of genes involved in *T*. *dichotomus* head horn development, we performed RNAi analysis in the red flour beetle *T*. *castaneum*. Although *T*. *castaneum* is a member of the Tenebrionidae, and not a direct ancestor of the Scarabaeidae, this beetle is considered to represent the ancestral head shape [24]. In addition, we used this species for comparison because of the availability of both genome sequence and the ability to easily perform systemic RNAi [33, 34]. We analyzed orthologs of the *T*. *dichotomus* clypeolabrum patterning genes we identified, *Rx*, *Optix*, *Tbx20* and *SP8*, and appendage-patterning genes, *BarH1*, *dac*, *Sox14* and *ab*.

Contrary to the drastic change in horn and clypeolabral morphologies in *T*. *dichotomus*, *Rx* RNAi caused no detectable changes to adult head morphology compared to control injections in *T*. *castaneum* (S7A and S7B Fig; n = 22 and 20 for *EGFP* and *Rx*, respectively). We could find all three SP family gene orthologs in the *T*. *castaneum* genome, which enabled us to target the *SP8* gene specifically [17] (S4 Fig). RNAi treatment for *SP8* in *T*. *castaneum* affected appendages as previously reported [16]. *SP8* RNAi resulted in fused appendage segments similar to results seen in *T*. *dichotomus*. (S7I Fig; n = 22). Notably, after *SP8* RNAi, the clypeolabral region of the flour beetle was unaffected (S7F–S7H Fig). In fact, we detected no obvious morphological changes in the head surface of *T*. *castaneum* after RNAi for either *Optix* or *Tbx20* (S7C and S7D Fig; n = 22 and 18 for *Optix* and *Tbx20*, respectively). We note that *Optix* RNAi affected compound eye formation, as has been reported (S7E Fig; t = 7.69, degree of freedom = 42, P = 1.53e^-09^, student’s t-test) [6].

These results suggest that the clypeolabrum patterning genes we examined, although critical for formation of the embryonic head, no longer exert detectible effects when knocked down in late-stage larval *T*. *castaneum*. This constitutes an important difference between head patterning in *Tribolium* flour beetles and both *Trypoxylus* rhinoceros beetles and *Onthophagus* dung beetles, and suggests that the origin of head horns in beetles may involve heterochronic shifts in the timing of patterning of the clypeolabral region. In contrast to the non-conserved function of clypeolabral patterning genes, the function of appendage patterning genes was well conserved between *T*. *dichotomus* and *T*. *castaneum*, as we observed similar effects between both species for all five genes (Fig 5G–5L; S7I Fig).

## Discussion

### Developmental origin of horns in *T*. *dichotomus*

**Fig 6:**
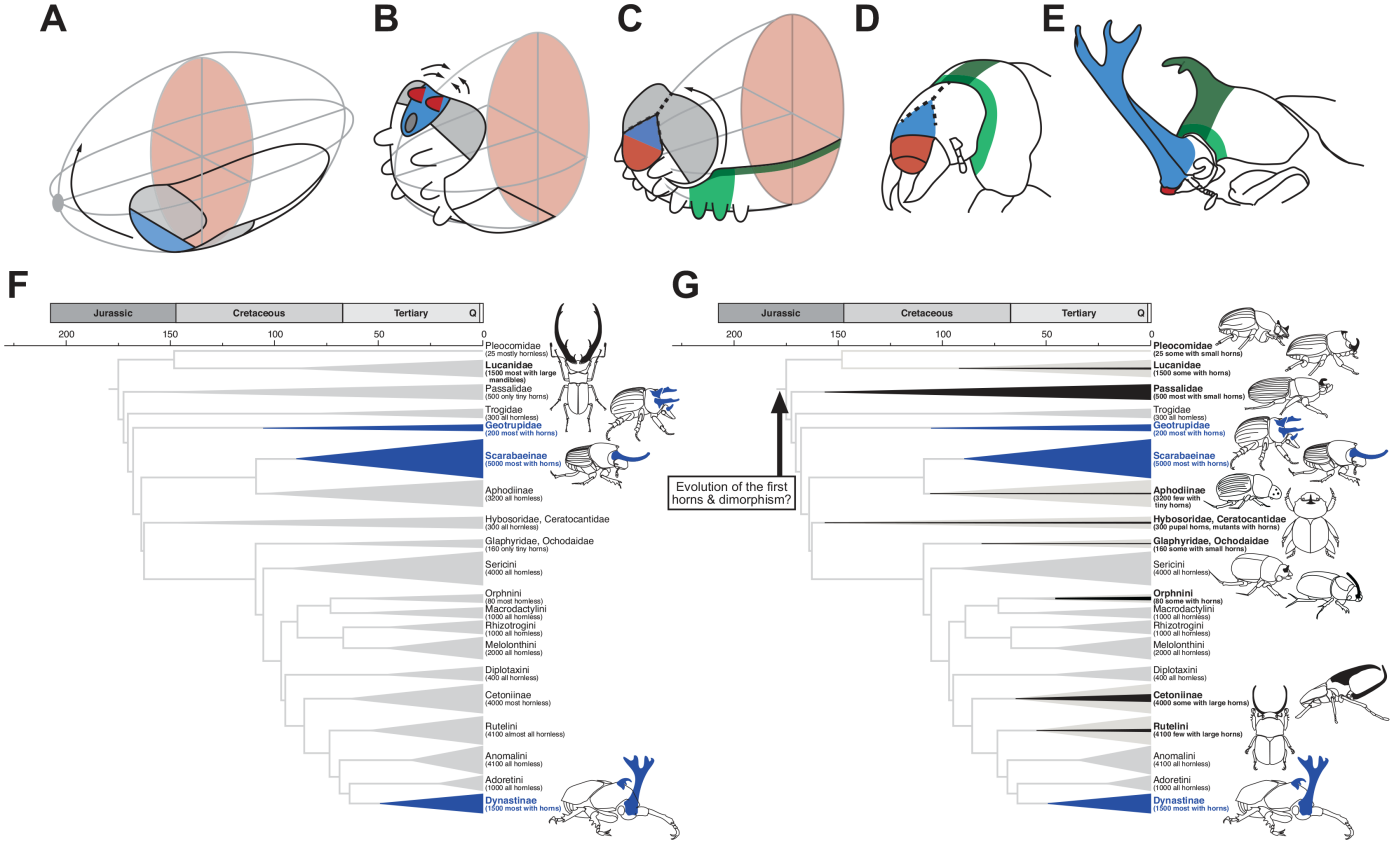
Developmental and evolutionary origin of head and thorax horns in Scarab beetles. (**A**–**C)** “Bend and zipper” model of head development in *T*. *castaneum* from Posnien *et al*. (2010) [35]. The clypeolabral region (light blue) lies anterior to the mouth and is originally part of the “pre-segmental” region of the germ band (**A**); This region migrates anteriorally and vertically, inverting and folding backwards and becoming enframed by the lateral head lobes (gray) (**B**) which fuse along the midline of the head (**C**). The labrum (red) is formed from the fusion of a pair of non-appendicular ventral evaginations in the clypeolabral region. (**D**, **E**) Head and prothorax fate map in larval *T*. *castaneum* (**D**) and inferred fate map in adult *T*. *dichotomus* (**E**) based on gene expression and RNAi knockdown. We propose that the head horn originated from the clypeolabral region (light blue), explaining our finding that ventral appendage patterning genes and clypeolabral genes function in horn growth even though it extends from the top of the head. Thoracic horns appear to arise from the dorsal midline at the juncture of the head and thorax, a region defined by both *Scr* (light green) and *pnr* (dark green) expression. Coronal and frontal sutures are shown in dotted lines in **C** and **D**. (**F)** Partial phylogeny for the families and subfamilies of scarab beetles (Coleoptera: Scarabaeidae), showing the presence of head and/or thoracic horns. The majority of species with exaggerated horns are concentrated within three distantly related clades (Geotrupidae, Scarabaeinae, Dynastinae) that collectively represent only 20% of extant scarab species. For this reason, horns are thought to have arisen multiple times independently within the superfamily (blue shading), leading Darwin and others to speculate on the “special tendency” of the scarabs towards evolution of enlarged horns. **(G)** However, eleven of the included clades contain either rudimentary horns, at least a few genera or species with enlarged horns, or mutant individuals with horns (black shading), and our present results reveal striking similarities in the development of Scarabaeinae and Dynastinae horns. Thus, an alternative explanation of our results is that the ancestor to the scarabs had both horns, and mechanisms for suppressing horn growth (e.g., sexual dimorphism). If true, then the repeated, parallel evolution of horns within the scarabaidae would reflect taxic atavism of an earlier ancestral state. Tree topology is derived from Ahrens *et al*., (2014) [7], but see also [71,72]; species numbers and taxon descriptions are derived from Ratcliffe and Jameson (2006) [73].

Recent advances in developmental genetics have elevated the flour beetle *T*. *castaneum* to become a model system for studying development in insects generally [35, 36]. In particular, studies of embryonic expression of trunk and appendage patterning genes recently led to a new model for the formation of segments and sutures in the insect head [35]. This “bend and zipper” model proposes that the flat epithelial band containing head segments and ventral appendage primordia migrates anteriorly, folding upwards and backwards (Fig 6A and 6B) [37]. The anterior head lobes grow around the clypeolabral region, eventually fusing with each other along the anterior midline of the head. This now-inverted epithelium fuses with the maxillary and labial segment regions of the layer below, completing the head capsule (Fig 6C and 6D). The bend and zipper model accounts for the mysterious placement of frontal/ clypeolabral appendages in Paleozoic Euarthropoda, and for the coronal, frontal, and subgenal sutures demarcating head capsules of many insects [35, 38].

Importantly, this model also provides a basis for proposing the developmental origins of horns in beetles, possibly explaining the paradoxical finding of our study as well as studies of dung beetles in the genus *Onthophagus* [6], that head horns, though appearing to lie on the top, or dorsal region of the beetle head, in fact express genes typical of anterior “pre-segmental” (clypeolabral) body regions and ventral imaginal discs. For example, *Optix*, *Rx*, and *Tbx20*, genes whose expression is confined to the pre-segmental clypeolabral region in diverse bilaterians including *T*. *castaneum* [26], are expressed in head horn tissues in *Trypoxylus dichotomus* and appear functionally involved with specifying horn size and shape (Fig 3). Similarly, the zinc-finger transcription factor *Sp8* (*D*. *melanogaster Sp1*) is known to affect relative amounts of growth of ventral appendages in *D*. *melanogaster* [13], *T*. *castaneum* [16] and milkweed bugs [39], and we show that disruption of *Sp8* through RNAi knockdown is sufficient to induce formation of an ectopic head horn in *T*. *dichotomus* (Fig 2B and 2C).

Together these results suggest that the head horns in both *Onthophagus* and *Trypoxylus* beetles form from appendage-like outgrowths in the clypeolabral region of the head, a pocket of anterior (pre-segmental) cells with ventrally-patterned outgrowths that folds upwards and backwards during embryogenesis such that the novel appendages growing from this region extend vertically from the top of the head in adult beetles (Fig 6E). Whether this means that beetle head horns are homologous with the various non-appendicular clypeo-labral evaginations of fossil and extant panarthropods (e.g., primary antennae of onychophorans) remains to be investigated [38].

In *T*. *castaneum* and other insects, including *D*. *melanogaster*, the homeotic gene *Scr* is localized to the dorsal ridge, the anterior-most region of the body capable of having a dorsal fate (note the consistency of this expression pattern with the “bend and zipper” model of head development, Fig 6) [40]. The dorsal ridge forms the boundary of the head and thorax, and is comprised of regions of the maxillary and labial segments, as well as parts of the first thoracic (T1) segment (prothorax). Although *Scr* retains this regional specification in insects pre-dating the origin of wings (e.g., firebrats), its best-studied function in pterygote species is to repress growth of wing primordia in the prothorax [41]. RNAi knockdown of *Scr* leads to vestigial T1 wings on the prothorax of *D*. *melanogaster*, milkweed bugs, cockroaches, mealworm beetles, *T*. *castaneum* as well as *Onthophagus* beetles [21, 41–45]. Wasik et al. (2010) showed that *Scr* RNAi knockdown affected development of prothoracic, but not head horns, in *Onthophagus*, and we demonstrate here that *Scr* RNAi leads to reduced growth of prothoracic, but not head horns in *T*. *dichotomus* [21] (Fig 3O).

Similarly, *pnr*, a gene involved in embryonic dorsal closure in *D*. *melanogaster*, is typically expressed along the dorsal midline of the thorax and abdomen, where it acts as a selector gene specifying dorsal-medial identity to tissues including the heart [18, 19]. Here we show that *pnr* expression is necessary for growth of the prothoracic horn, as RNAi knockdown resulted in a complete loss of the dorsal-medial region of the thorax, including loss of the entire horn (Fig 2E). Together, these results suggest the developmental locus of the thoracic horn in *T*. *dichotomus* is the anteriormost zone of the dorsal midline, a region specified by intersecting domains of expression of *Scr* and *pnr* (a region defined by both light green and dark green in Fig 6C–6E).

### Evolutionary origin(s) of beetle horns

The Scarabaeidae contain approximately 35,000 species, the overwhelming majority of which are hornless. Yet, horns are thought to have arisen many times independently within this clade, such that today several thousand species bear elaborate weapons. The extreme sizes of these structures, and their concentration within a single family of beetles, led Darwin to conclude that sexual selection acted especially effectively in scarab beetles [46], and Arrow suggested they have a “special tendency” to the acquisition of horns [47]. Arrow went so far as to conclude “it is certain that these horns have had no common origin” [47]. Horns are assumed to have arisen multiple times for two reasons: most scarab species (80%) lack horns; and the sub-families with the majority of horned species (“dor” beetles [Geotrupinae]; dung beetles [Scarabaeinae]; rhinoceros beetles [Dynastinae]) are too widely dispersed within the Scarabaeidae (Fig 6F).

Our study provides the first detailed characterization of horn development from a rhinoceros beetle, *T*. *dichotomus*, permitting the parallel origins of rhinoceros and dung beetle horns to be contrasted at a mechanistic level. Although a few genes clearly show lineage specific differences in function (e.g., *dac* affects horns in *T*. *dichotomus* but not *Onthophagus*), the overwhelming pattern is one of similarity. Head horns in both lineages arise from ventral appendage-like outgrowths in the anteriormost, pre-segmental clypeolabral region of the head; in both lineages the expression and function of these clypeolabral patterning genes appear to involve a heterochronic shift markedly divergent from head development in *T*. *castaneum* (S7 Fig); and thoracic horn outgrowths in both lineages appear to extend from the anteriormost region of the dorsal midline, a zone specified by the homeotic gene *Scr*, and, we now show, the domain of expression of *pnr*.

In addition to the precise locations of horn outgrowth being similar, the formation of the outgrowths themselves appears similar, involving in both horn types and in both lineages the partial deployment of appendage patterning networks. Finally, recent studies of the mechanisms of sexual dimorphism in beetles suggest that sexually dimorphic growth of both types of horns is regulated by the same pathway. Alternative splice forms of *doublesex* in males and females regulate sex specific patterns of growth of enlarged mandibles in stag beetles (Lucanidae), as well as horns in *Onthophagus* and *Trypoxylus*, consistent with a shared capacity for female-specific repression of weapon growth across the scarabs [9, 48, 49]. Consequently, our results reveal many layers of mechanistic parallelism between the horns of rhinoceros and dung beetles (S8 Fig), and point to a surprisingly repeatable path to the evolution of these extreme, sexually selected structures.

An alternative explanation is that horns arose once, before the diversification of the scarabs, and that the repeated evolution of horns in diverse lineages represents “taxic atavism” [50, 51] as has been described recently for amphibian teeth [52] and supersoldier castes in *Pheidole* ants [53]. Indeed, several clues suggest the ancestral scarab beetles may have been horned. First, most of the primarily-hornless subfamilies contain at least a few species with either rudimentary horns (e.g., Pleocomidae, Passalidae, Ochodaidae, Orphninae) or with fully-developed horns (e.g., Melolonthinae, Cetoniinae, Rutelinae; Fig 6G). Second, the pupal stages of many scarabs have thoracic ‘horns’, and these are often present in individuals (e.g. females) or species that lack this horn as adults. Pupal ‘horns’ may serve a current function as support structures protecting animals during the vulnerable metamorphic molt [54, 55], but they may also represent developmental carry-overs from a horn that was present in the adult stages of an ancestor [56, 57]. Third, even within completely hornless species—in one case a species within a completely hornless subfamily, the Ceratocanthidae, which have been a distinct clade for at least 65 million years—mutant adult individuals occasionally appear with fully developed horns, and these horns also resemble the horns of other scarabs [56, 58] (Fig 6G). These observations led Emlen *et al*. (2006) to propose that perhaps the ancestral scarabs did have horns, as well as a developmental capacity to shut off horn growth (e.g., in females) [59]. If true, this would mean that the hornless state of most present-day scarab species reflects a derived condition entailing the repression of horn growth. Future studies will be needed to distinguish between these alternatives, including examining horn growth in additional horned scarab lineages such as the Cetoniidae and Geotrupidae, and testing whether the putative ancestral developmental potential to produce horns remains in currently hornless species.

## Materials and methods

### Beetle rearing

We purchased larvae of *T*. *dichotomus* from Kuwagata Koubo Mushikichi (Fukuoka, Japan), and Roiene (Gunma, Japan). Larvae were sexed as previously described [9], individually fed on humus in plastic containers, and kept at 10 °C until use.

*T*. *castaneum* was reared on flour powder in plastic containers at 30 °C. After dsRNA injections, each larva was separated in 24-well plates until adulthood.

### Sample collection, cDNA library construction and sequencing

Head and thoracic horn primordia were manually dissected out from *T*. *dichotomus* larvae in ice-cold 0.75% sodium chloride, snap-frozen in liquid nitrogen and stored at -80 °C until use. The developmental stage of each tissue was determined from the external morphology of dissected horn primordia. Total RNA was extracted using the RNeasy mini kit (Qiagen, Valencia, CA, USA) according to manufacturer’s instruction. On-column DNase I treatment was performed. RNA purity was assessed with Qubit RNA HS assay kit (Thermo Fischer Scientific, MA, USA). RNA integrity was analyzed on a Bioanalyzer 2100 (Agilent Technologies, CA, USA), and RNA quality is shown in S8 Table. One μg of total RNA from a single beetle was used for each paired-end cDNA library construction using the TruSeq RNA sample preparation kit (Illumina, San Diego, CA, USA). Three biological replicates were sequenced for each sample. The cDNA library was sequenced on an Illumina HiSeq 2000 (Illumina, San Diego, CA) generating 150 bp paired-end reads.

### De novo transcript assembly and sequence analysis

We performed *de novo* assembly of short read sequences using Trinity (version r2012-06-08) with default configurations without any additional options [60]. Raw RNA-seq data and the assembled transcripts are deposited in DDBJ Sequence Read Archive under project accession number PRJDB6456, and in DDBJ Transcriptome Shotgun Assembly division under accession numbers IADJ01000001-IADJ01127986 (127986 entries), respectively. The BLASTnr version released on Oct 30, 2012 was used for annotation of the transcript model. The same version of BLASTnr, and OrthoDB 5 databases were used for the qualification of transcriptome shown in S1 Fig. Cutoff e-values for the BLAST searches against these databases were 1.0e-4. Both Metazoa ODB9 and Insecta ODB9 datasets were used for BUSCO analysis [11]. We performed read counting and differential expression analysis using RSEM (version 1.1.21) with default configurations [61]. Multidimensional scaling of raw count values was performed and visualized with R (version 3.3.3 (2017-03-06)) [62]. For identification of differentially expressed genes (DEGs) between samples, we used the TCC package with default options, and multi-step normalization and edgeR-based DEG analysis. In this strategy, normalization of count data and DEG estimation are iterated for avoiding false positives, and we repeated this cycle three times in this study [63–65]. DEGs between samples were defined as genes at false discovery rate lower than 0.05. For MA plots in Fig 1, M and A values were calculated using TCC package, and visualized with R (version 3.3.3 (2017-03-06)) [62, 65]. Raw numerical data for the DEG analysis are provided as S1–S4 Appendices. As several new methods have been developed after our initial analysis, we now include summaries of sequence data analyses with these alternative methods, which include analysis using a newer version of Trinity, and with tag quantification with Kallisto and Salomon, in S7 Appendix [60, 66, 67].

### Sp family gene phylogenetic analysis

Alignment and phylogenetic neighbor-joining (NJ) tree of SP family genes was constructed by Clustal X using putative amino acid sequences [68].

### Gene ontology (GO) enrichment analysis

We searched orthologous genes to the assembled transcripts from OrthoDB database. GO terms were assigned to assembled transcripts based on GO information on orthologous genes found in the FlyBase database. We then analyzed GO terms over-represented in each comparison between RNA-seq data set using ErmineJ (version 3.0.2) [12]. GO terms enriched at an FDR < 0.5 for each comparison in ErmineJ anlaysis were summarized with REVIGO and visualized with R (version 3.3.3 (2017-03-06)) [62, 69].

### RNAi-mediated gene knockdown

953 bp, 482 bp and 841 bp cDNA fragments of *esg*, *B-H1* and *dac* were first subcloned into the plasmid pCR4-TOPO (Invitrogen), respectively. We *in vitro* transcribed double-stranded RNAs (dsRNAs) for these genes from purified PCR products using primers 5´-TAATACGACTCACTATAGGGAGACCACGTCCTGCAGGTTTAAACG-3´ and 5´-TAATACGACTCACTATAGGGAGACCACCGAATTGAATTTAGCGGC-3´.

For the remaining genes, first-stranded cDNA (fs cDNA) was synthesized with the SuperScript III Reverse Transcriptase (Thermo Fischer Scientific, MA, USA) using one μg total RNA extracted from head and prothoracic horn primordia of three males and three females at the prepupal stage. Sample collection and total RNA extraction were performed as described above. Equal amounts of each fs cDNA were mixed and used as a template for PCR. The PCR was performed using gene specific primers with T7 sequence at 5´ end listed in S9 Table, and *in vitro* transcribed RNAs were made from purified PCR products using the AmpliScribe T7-Flash Transcription Kit (Epicentre, WI, USA). These gene-specific primers were designed in open reading frames to produce a product of 300–400 bp length, for which the target specificity was confirmed by BLAST search against the transcriptome. We ensured that the expected size of a PCR product was amplified on an agarose gel, cut a single band from the gel, and purified the product prior to *in vitro* transcription. A single dsRNA was used for each target gene, and a dsRNA targeting *EGFP* gene was used as a control.

We injected dsRNA into the hemocoel of last instar larvae through the intersegmental membrane at the anterior-lateral position of the prothoracic segment using a syringe (Terumo Corporation, Tokyo, Japan) with a 30-gauge needle (Becton, Dickinson and Company, NJ, USA). Last instar beetle larvae were moved from 10°C to room temperature (about 25 °C) at least 2 days before dsRNA injection, and reared at room temperature after the injection until they developed into adults. Our timing of dsRNA injection preceded the onset of horn formation, as larvae normally form pupal chambers several days after moving from 10°C to room temperature, and development of sexually dimorphic horns begins during prepupal period after pupal chamber formation [70]. We note that beetles need to be stored at 10 °C as the supply is seasonal, and that storage duration of larvae at low temperature might influence the survival rate of injected beetles. The data shown include all experiments performed on both short-stored and long-stored larvae.

For *T*. *castaneum*, dsRNA was injected into late larvae using glass needles with a Femtojet (Eppendolf, Hamburg, Germany). We injected approximately 0.83 μg of dsRNAs into each larva. We repeated the experiment for *Rx*, *Tbx20* and *SP8* because we did not find any change after the first injections for *Rx* and *Tbx20*, and because a sufficient number of beetles were unable to eclose in *SP8* RNAi. For this second experiment, the amount of dsRNAs were changed to approximately 3.32 μg for *Rx* and *Tbx20*, and 0.083 μg for *SP8*.

### Horn area and length measurement

Horn area was measured in pictures of each horn region (i.e. central and side grooves in head horn, and thoracic horn groove) on a flat surface by using ImageJ 64. For the side grooves on the head horn, two areas were separately measured and averaged. Horn length was analyzed from image data of either heads or prothoraxes separated from the other body segments and imaged from the lateral aspect. Both dorsal and ventral length were separately measured using the “SegmentMeasure” plug-in for ImageJ 64 developed by Hosei Wada. Note that we included non-horn body wall parts to obtain “horn length” data in order to measure corresponding segments among different dsRNA treated animals. Dorsal and ventral length were summed after values from right and left views were averaged. Body length, from the anterior tip of clypeus to the posterior most region of the body, was measured with a digital caliper model DN-100 (Niigata seiki, Co., Ltd., Niigata, Japan). We utilized logistic regression and a Wald test in R (version 3.3.3 (2017-03-06)) to test for significance [62]. We omitted some eclosed individuals from the measurement because we were unable to apply this scheme for measurement due to highly malformed horns. Although the number of available larvae from the supplier is limited, we analyzed at least five surviving individuals per gene. Raw numerical data for the area and length measurements are provided as S5 and S6 Appendices, respectively.

### Microscopy

Photographs were taken with digital microscopes; either the VHX-900 or VHX-5000 (KEYENCE, Co., Osaka, Japan). Scanning electron micrograph were taken with a VHX-D500 (KEYENCE, Co., Osaka, Japan). Adobe Photoshop CS5.1 and Adobe Illustrator CS5.1 (Adobe Systems, Inc., San Jose, CA) were used for image processing and assembly.

## Supporting information

S1 Fig Similarity of the assembled transcript to public sequences. (A—C) Similarity search to NCBI nr database. The proportion of assembled transcripts that show the highest similarity to each group is indicated in phylum (A), order (B) and species (C) levels. (D and E) Similarity search to the fruit fly *Drosophila melanogaster* (D) and the red flour beetle *Tribolium castaneum* (E) sequences in OrthoDB5. The number of transcripts or genes belonging to each section and the percentage of *T*. *dichotomus* transcripts that have putative orthologous genes in OrthoDB5 database are indicated. (F) Results of BUSCO analysis against either Metazoa or Insecta database are shown.
(PDF)

S2 Fig Count data is distinctly distributed among samples. A multi-dimentional scaling plot of count data.
(PDF)

S3 Fig Developmental genes are differentially expressed between head horn and thoracic horn. Enriched GO terms in each data set comparison that clustered based on semantic similarity of GO terms using REVIGO. The size of each point reflects the number of genes assigned to a GO term. Color indicates enrichment false discovery rate (FDR) in GO enrichment analysis using ErmineJ. GO terms enriched at FDR < 0.5 for each comparison in ErmineJ anlaysis are plotted. Descriptions of 10 GO terms that have the lowest FDR are shown on each plot.
(JPG)

S4 Fig Molecular phylogeny of SP family genes. A phylogenetic tree (A) and multiple amino acid sequence alignment (B) of SP family genes in representative insects. The number at nodes indicate boot strap values in A. Amino acid identity and similarity among sequences are indicated by asterisks and dots, respectively.
(JPG)

S5 Fig Sox14 RNAi beetle showed defects in overall segment formation. Dorsal view of adult male beetles. Scale bar is 1 cm.
(TIF)

S6 Fig RNAi for *BarH1*, *Sox21b*, *dac*, *Optix* and *Tbx20* caused no reduction in horn length. Lateral view of head and prothorax in RNAi beetles are displayed. Horn length and body length are plotted, and linear regression lines are drawn for each gene in red. Gray dots and gray regression lines are for *EGFP* RNAi beetles. P-values show significance of each RNAi treatment using Wald test on logistic linear regression.
(TIF)

S7 Fig RNAi caused differential effects on head, but similar effects on appendage between *Tribolium castaneum* and *Trypoxylus dichotomus*. (A—D) Dorsal view of *T*. *castaneum* adult heads after RNAi treatments. (E) A box plot shows decreased number of ommatidium in *Optix* RNAi beetle compared to control. (F) Dorsal view of an *SP8* RNAi beetle. Arrowheads in F indicate malformed antennae in *SP8* RNAi beetles. (G and H) Frontal view of adult heads in *EGFP* (G) and *SP8* (H) RNAi beetles. Arrowheads point to corresponding regions between G and H, which is fused by *SP8* RNAi treatment (H). The dorsal part of the labrum is in red. (I) Adult metathoracic legs in *T*. *castaneum* after RNAi treatments. Scale bar is 0.1 mm in A—D, F—H, 0.5 mm in I.
(TIF)

S8 Fig Comparison of mechanisms regulating head and thoracic horn growth in *Trypoxylus dichotomus* and *Onthophagus spp*. Both thoracic (A) and head (B) horns appear to have arisen through the repeated evolutionary co-option of parallel mechanistic processes, including the pathway regulating sexually dimorphic amounts of weapon growth (alternative splice forms of *doublesex*), the embryonic locations of horn outgrowth, and the co-option of portions of the insect appendage patterning pathway. However, the specific genes *within* the patterning pathway with the most pronounced effects on horn size differ somewhat between rhinoceros and dung beetle horns, consistent with their presumed independent evolutionary origins. Results summarized from [2], [5–6], [9–10], [21], [49].
(JPG)

S1 Table Summary of sequencing and de novo transcript assembly. (PDF)

S2 Table Number of differentially expressed genes with a cut off value of FDR < 0.05. (PDF)

S3 Table Gene set analysis. (PDF)

S4 Table Summaruy of RNAi analysis. (PDF)

S5 Table Wald test for RNAi effects on horn shape. (PDF)

S6 Table Wald test for RNAi effects on horn length. (PDF)

S7 Table BDGP terms and function in appendages. (PDF)

S8 Table Total RNA quality. (PDF)

S9 Table Primers. (PDF)

S1 Appendix Result of DEG analysis: Male head horn vs female head horn. (TXT)

S2 Appendix Result of DEG analysis: Male thoracic horn vs female thoracic horn. (TXT)

S3 Appendix Result of DEG analysis: Male head horn vs male thoracic horn. (TXT)

S4 Appendix Result of DEG analysis: Female head horn vs female thoracic horn. (TXT)

S5 Appendix Raw data for area measurements. (CSV)

S6 Appendix Raw data for length measurements. (CSV)

S7 Appendix Summary of alternative data analyses. (DOCX)

## References

[1] Moczek AP. On the origins of novelty in development and evolution. BioEssays. 2008; 30: 432–447. 10.1002/bies.20754 18404691

[2] Moczek AP, Nagy LM. Diverse developmental mechanisms contribute to different levels of diversity in horned beetles Evol Dev. 2005; 7: 175–185. 10.1111/j.1525-142X.2005.05020.x 15876190

[3] Emlen DJ, Lavine CL, Ewen-Campen B. On the origin and evolutionary diversification of beetle horns Proc Natl Acad Sci U S A. 2007; 104 (Suppl): 8661–8668. 17494751 10.1073/pnas.0701209104 PMC1876444

[4] Matsuda K, Gotoh H, Tajika Y, Sushida T, Aonuma H, Niimi T, et al Complex furrows in a 2D epithelial sheet code the 3D structure of a beetle horn. Sci Rep. 2017; 7: 13939 10.1038/s41598-017-14170-w 29066748 PMC5655322

[5] Moczek AP, Rose DJ. Differential recruitment of limb patterning genes during development and diversification of beetle horns. Proc Natl Acad Sci U S A. 2009; 106: 8992–8997. 10.1073/pnas.0809668106 19451631 PMC2690047

[6] Zattara EE, Busey HA, Linz DM, Tomoyasu Y, Moczek AP. Neofunctionalization of embryonic head patterning genes facilitates the positioning of novel traits on the dorsal head of adult beetles. Proc R Soc B. 2016; 283: 20160824 10.1098/rspb.2016.0824 27412276 PMC4947891

[7] Ahrens D, Schwarzer J, Vogler AP. The evolution of scarab beetles tracks the sequential rise of angiosperms and mammals. Proc R Soc B. 2014; 281: 20141470 10.1098/rspb.2014.1470 25100705 PMC4132691

[8] Emlen DJ, Marangelo J, Ball B, Cunningham CW. Diversity in the weapons of sexual selection: horn evolution in the beetle genus *Onthophagus* (Coleoptera: Scarabaeidae). Evolution. 2005; 59: 1060–1084. 16136805

[9] Ito Y, Harigai A, Nakata M, Hosoya T, Araya K, Oba Y, et al The role of *doublesex* in the evolution of exaggerated horns in the Japanese rhinoceros beetle. EMBO Rep. 2013; 14: 561–567. 10.1038/embor.2013.50 23609854 PMC3674438

[10] Kijimoto T, Andrews J, Moczek AP. Programed cell death shapes the expression of horns within and between species of horned beetles. Evol Dev. 2010; 12: 449–458. 10.1111/j.1525-142X.2010.00431.x 20883214

[11] Simão FA, Waterhouse RM, Ioannidis P, Kriventseva EV, Zdobnov EM. BUSCO: Assessing genome assembly and annotation completeness with single-copy orthologs. Bioinformatics. 2015; 31: 3210–3212. 10.1093/bioinformatics/btv351 26059717

[12] Lee HK, Braynen W, Keshav K, Pavlidis P. ErmineJ: Tool for functional analysis of gene expression data sets. BMC Bioinformatics. 2005; 6: 269 10.1186/1471-2105-6-269 16280084 PMC1310606

[13] Estella C, Rieckhof G, Calleja M, Morata G. The role of *buttonhead* and *Sp1* in the development of the ventral imaginal discs of *Drosophila*. Development. 2003; 130: 5929–5941. 10.1242/dev.00832 14561634

[14] Estella C, Mann RS. Non-Redundant Selector and Growth-Promoting Functions of Two Sister Genes, buttonhead and Sp1, in Drosophila Leg Development. PLoS Genet. 2010; 6: e1001001 10.1371/journal.pgen.1001001 20585625 PMC2891808

[15] Schöck F, Purnell BA, Wimmer EA, Jäckle H. Common and diverged functions of the *Drosophila* gene pair *D-Sp1* and *buttonhead*. Mech Dev. 1999; 89: 125–132. 10559487 10.1016/s0925-4773(99)00215-4

[16] Beermann A, Aranda M, Schröder R. The *Sp8* zinc-finger transcription factor is involved in allometric growth of the limbs in the beetle *Tribolium castaneum*. Development. 2004; 131: 733–742. 10.1242/dev.00974 14724124

[17] Schaeper ND, Prpic N, Wimmer EA. A clustered set of three Sp-family genes is ancestral in the Metazoa: evidence from sequence analysis, protein domain structure, developmental expression patterns and chromosomal location. BMC Evol Biol. 2010; 10: 88 10.1186/1471-2148-10-88 20353601 PMC3087555

[18] Heitzler P, Haenlin M, Ramain P, Cauejat M, Simpson PA. Genetic analysis of *pannier*, a gene necessary for viability of dorsal tissues and bristle positioning in *Drosophila*. Genetics. 1996; 143: 1271–1286. 8807299 10.1093/genetics/143.3.1271 PMC1207396

[19] Herranz H, Morata G. The functions of *pannier* during *Drosophila* embryogenesis. Development. 2001; 128: 4837–4846. 11731463 10.1242/dev.128.23.4837

[20] Hughes CL, Kaufman TC. *Hox* genes and the evolution of the arthropod body plan. Evol Dev. 2002; 4: 459–499. 12492146 10.1046/j.1525-142x.2002.02034.x

[21] Wasik BR, Rose DJ, Moczek AP. Beetle horns are regulated by the *Hox* gene, *Sex combs reduced*, in a species- and sex-specific manner. Evol Dev. 2010; 12: 353–362. 10.1111/j.1525-142X.2010.00422.x 20618431

[22] Ritter AR, Beckstead RB. Sox14 is required for transcriptional and developmental responses to 20-hydroxyecdysone at the onset of *Drosophila* metamorphosis. Dev Dyn. 2010; 239: 2685–2694. 10.1002/dvdy.22407 20803583

[23] Lyne R, Smith R, Rutherford K, Wakeling M, Varley A, Guillier F, et al FlyMine : an integrated database for *Drosophila* and *Anopheles* genomics. Genome Biol. 2007; 8: R29. 17615057 10.1186/gb-2007-8-7-r129 PMC2323218

[24] Posnien N, Bashasab F, Bucher G. The insect upper lip (labrum) is a nonsegmental appendage-like structure. Evol Dev. 2009; 11: 480–488. 10.1111/j.1525-142X.2009.00356.x 19754705

[25] Scholtz G., Edgecombe G. The evolution of arthropod heads: reconciling morphological, developmental and palaeontological evidence. Dev Genes Evol. 2006; 216: 395–415. 10.1007/s00427-006-0085-4 16816969

[26] Posnien N, Koniszewski NDB, Hein HJ, Bucher G. Candidate gene screen in the red flour beetle *Tribolium* reveals *Six3* as ancient regulator of anterior median head and central complex development. PLoS Genet. 2011; 7: e1002416 10.1371/journal.pgen.1002416 22216011 PMC3245309

[27] Wimmer EA, Jackle H, Pfeifle C, Cohen SM. A *Drosophila* homologue of human *Sp1* is a head-specific segmentation gene. Nature. 1993; 363: 690–694. 10.1038/366690a0 8259212

[28] Diaz-Benjumea FJ, Cohen B, Cohen SM. Cell interaction between compartments establishes the proximal-distal axis of *Drosophila* legs. Nature. 1994; 372: 175–179. 10.1038/372175a0 7969450

[29] Zecca M, Basler K, Struhl G. Sequential organizing activities of engrailed, hedgehog and decapentaplegic in the *Drosophila* wing. Development. 1995; 121: 2265–2278. 7671794 10.1242/dev.121.8.2265

[30] Kojima T, Sato M, Saigo K. Formation and specification of distal leg segments in *Drosophila* by dual Bar homeobox genes, *BarH1* and *BarH2*. Development. 2000; 127: 769–778. 10648235 10.1242/dev.127.4.769

[31] Mardon G, Solomon NM, Rubin GM. *dachshund* encodes a nuclear protein required for normal eye and leg development in *Drosophila*. Development. 1994; 120: 3473–3486. 7821215 10.1242/dev.120.12.3473

[32] Hu S, Fambrough D, Atashi JR, Goodman CS, Crews ST. The *Drosophila abrupt* gene encodes a BTB-zinc finger regulatory protein that controls the specificity of neuromuscular connections. Genes Dev. 1995; 9: 2936–2948. 7498790 10.1101/gad.9.23.2936

[33] Tribolium Genome Sequencing Consortium. The genome of the model beetle and pest *Tribolium castaneum*. Nature. 2008; 452: 949–955. 10.1038/nature06784 18362917

[34] Tomoyasu Y, Denell RE. Larval RNAi in *Tribolium* (Coleoptera) for analyzing adult development. Dev Genes Evol. 2004; 214: 575–578. 10.1007/s00427-004-0434-0 15365833

[35] Posnien N, Schinko JB Kittelmann S, Bucher G. Genetics, development and composition of the insect head—A beetle’s view. Arthropod Struct Dev. 2010; 39: 399–410. 10.1016/j.asd.2010.08.002 20800703

[36] Schmitt-Engel C, Schultheis D, Schwirz J, Strohlein N, Troelenberg N, Majumdar U, et al The iBeetle large-scale RNAi screen reveals gene functions for insect development and physiology. Nat Commun. 2015; 6: 7822 10.1038/ncomms8822 26215380 PMC4525174

[37] Posnien N, Bucher G. Formation of the insect head involves lateral contribution of the intercalary segment, which depends on *Tc-labial* function. Dev Biol. 2010; 338: 107–116. 10.1016/j.ydbio.2009.11.010 19913530

[38] Budd GE, Ortega-hern J. The nature of non-appendicular anterior paired projections in Palaeozoic total-group Euarthropoda. Arthropod Struct Dev. 2016; 45: 185–199. 10.1016/j.asd.2016.01.006 26802876

[39] Schaeper ND, Prpic NM, Wimmer EA. A conserved function of the zinc finger transcription factor Sp8/9 in allometric appendage growth in the milkweed bug *Oncopeltus fasciatus*. Dev Genes Evol. 2009; 219: 427–435. 10.1007/s00427-009-0301-0 19760183 PMC2773111

[40] Shippy TD, Rogers CD, Beeman RW, Brown SJ, Denell RE. The *Tribolium castaneum* ortholog of *Sex combs reduced* controls dorsal ridge development. Genetics. 2006; 174: 297–307. 10.1534/genetics.106.058610 16849608 PMC1569817

[41] Rogers BT, Peterson MD, Kaufman TC. Evolution of the insect body plan as revealed by the Sex combs reduced expression pattern. Development. 1997; 124: 149–157. 9006076 10.1242/dev.124.1.149

[42] Chesebro J, Hrycaj S, Mahfooz N, Popadic A. Diverging functions of Scr between embryonic and post-embryonic development in a hemimetabolous insect, *Oncopeltus fasciatus*. Dev Biol. 2009; 329: 142–151. 10.1016/j.ydbio.2009.01.032 19382295 PMC2775506

[43] Hrycaj S, Chesebro J, Popadic A. Functional analysis of Scr during embryonic and post-embryonic development in the cockroach, *Periplaneta americana*. Dev Biol. 2010; 341: 324–334. 10.1016/j.ydbio.2010.02.018 20171962 PMC2856087

[44] Ohde T, Yaginuma T, Niimi T. Insect morphological diversification through the modification of wing serial homologs. Science. 2013; 340: 495–498. 10.1126/science.1234219 23493422

[45] Tomoyasu Y, Wheeler SR, Denell RE. *Ultrabithorax* is required for membranous wing identity in the beetle *Tribolium castaneum*. Nature. 2005; 433: 643–647. 10.1038/nature03272 15703749

[46] Darwin CR. The descent of man, and selection in relation to sex. John Murray; 1871.

[47] Arrow GJ. Horned beetles: A study of the fantastic in nature. Junk W, The Hague; 1951.

[48] Gotoh H, Miyakawa H, Ishikawa A, Ishikawa Y, Sugime Y, Emlen DJ, et al Developmental link between sex and nutrition; *doublesex* regulates sex-specific mandible growth via juvenile hormone signaling in stag beetles. PLoS Genet. 2014; 10: e1004098 10.1371/journal.pgen.1004098 24453990 PMC3894178

[49] Kijimoto T, Moczek AP, Andrews J. Diversification of *doublesex* function underlies morph-, sex-, and species-specific development of beetle horns. Proc Natl Acad Sci U S A. 2012; 109: 20526–20531. 10.1073/pnas.1118589109 23184999 PMC3528601

[50] West-Eberhard M. J. Developmental plasticity and evolution. Oxford University Press; 2003.

[51] Abouheif E, Favé MJ, Ibarrarán-Viniegra AS, Lesoway MP, Rafiqi AM, Rajakumar R. Eco-evo-devo: the time has come In Ecological genomics (pp. 107–125). Springer, Dordrecht; 2014. 10.1007/978-94-007-7347-9_6 24277297

[52] Wiens JJ. Re-evolution of lost mandibular teeth in frogs after more than 200 million years, and re-evaluating Dollo’s law. Evolution. 2011; 65: 1283–1296. 10.1111/j.1558-5646.2011.01221.x 21521189

[53] Rajakumar R, San Mauro D, Dijkstra MB, Huang MH, Wheeler DE, Hiou-Tim F, et al Ancestral developmental potential facilitates parallel evolution in ants. Science. 2012; 335: 79–82. 10.1126/science.1211451 22223805

[54] Main H. Notes on the metamorphosis of *Onthophagus taurus* L. Proc Entom Soc London. 1922; 1922: 14–16.

[55] Edmonds WD, Hallfter G. Taxonomic review of immature dung beetles of the subfamily Scarabaeinae (Coleoptera: Scarabaeidae). Syst Entomol. 1978; 3: 307–331.

[56] Ballerio A. Revision of the genus Pterorthochaetes, first contribution (Coleoptera: Scarabaeoidea: Ceratocanthidae). Folia Heyrovskiana. 1999; 7: 221–228.

[57] Moczek AP. Pupal remodeling and the development and evolution of sexual dimorphism in horned beetles. Am Nat. 2006; 168: 711–729. 10.1086/509051 17109315

[58] Ziani S. Un interesante caso di teratologia simmetrica in *Onthophagus* (Paleonthophagus) *fracticornis* (Coleoptera: Scarabaeidae). Bolletino dell’Associazione Romana di Entomologia. 1995; 49: 3–4.

[59] Emlen DJ, Szafran Q, Corley LS, Dworkin I. Insulin signaling and limb-patterning: candidate pathways for the origin and evolutionary diversification of beetle ‘horns’. Heredity. 2006; 97: 179–191. 10.1038/sj.hdy.6800868 16850039

[60] Grabherr MG, Haas BJ, Yassour M, Levin JZ, Thompson D, Amit I, et al Full-length transcriptome assembly from RNA-seq data without a reference genome. Nat Biotechnol. 2011; 15: 644–652. 10.1038/nbt.1883 PMC3571712 21572440

[61] Li B, Dewey CN. RSEM: accurate transcript quantification from RNA-Seq data with or without a reference genome. BMC Bioinformatics. 2011; 12: 323 10.1186/1471-2105-12-323 21816040 PMC3163565

[62] R Core Team. R: A language and environment for statistical computing. R Foundation for Statistical Computing, Vienna, Austria 2017 https://wwwR-projectorg/

[63] Robinson MD, Oshlack A. A scaling normalization method for differential expression analysis of RNA-seq data. Genome Biol. 2010; 11: R25 10.1186/gb-2010-11-3-r25 20196867 PMC2864565

[64] Robinson MD, McCarthy DJ, Smyth GK. edgeR: a Bioconductor package for differential expression analysis of digital gene expression data. Bioinformatics. 2010; 26: 139–140. 10.1093/bioinformatics/btp616 19910308 PMC2796818

[65] Sun J, Nishiyama T, Shimizu K, Kadota K. TCC: An R package for comparing tag count data with robust normalization strategies. BMC Bioinformatics. 2013; 14: 219 10.1186/1471-2105-14-219 23837715 PMC3716788

[66] Bray NL, Pimentel H, Melsted P, Pachter L. Near-optimal probabilistic RNA-seq quantification. Nature Biotechnol. 2016; 34: 525–527. 27043002 10.1038/nbt.3519

[67] Patro R, Duggal G, Love MI, Irizarry RA, Kingsford C. Salmon provides fast and bias-aware quantification of transcript expression. Nat Methods. 2017; 14: 417–419. 10.1038/nmeth.4197 28263959 PMC5600148

[68] Thompson JD, Gibson T, Higgins DG. Multiple sequence alignment using ClustalW and ClustalX. Curr Protoc Bioinformatics. 2002; 00: 2.3.1–2.3.22. 10.1002/0471250953.bi0203s00 18792934

[69] Supek F, Bosnjak M, Skunca N, Smuc T. REVIGO summarizes and visualizes long lists of Gene Ontology terms. PLoS ONE. 2011; 6: e21800 10.1371/journal.pone.0021800 21789182 PMC3138752

[70] Morita S, Ando T, Maeno A, Mizutani T, Mase M, Shigenobu S, et al doublesex regulates sexually dimorphic beetle horn formation by integrating spatial and temporal developmental contexts in the Japanese rhinoceros beetle Trypoxylus dichotomus. bioRxiv. 2018; 10.1101/328120.

[71] Smith AB, Hawks DC, Heraty JM. An overview of the classification and evolution of the major scarab beetle clades (Coleoptera: Scarabaeoidea) based on preliminary molecular analyses. The Coleopterists Bulletin. 2006; 60: 35–46.

[72] Mckenna DD, Farrell BD, Caterino MS, Farnum CW, Hawks DC, Maddison DR, Seago AE, Short AE, Newton AF, Thayer MK. Phylogeny and evolution of Staphyliniformia and Scarabaeiformia: forest litter as a stepping stone for diversification of nonphytophagous beetles. Systematic Entomology. 2015; 40:35–60.

[73] Ratcliffe BC., Jameson ML. (eds) Generic guide to new world scarabs. 2006. http://museum.unl.edu/research/entomology/Guide/Guide-introduction/Guideintro.html

