# Supplementary figures and images for: Rhinoceros beetle horn development reveals deep parallels with dung beetles
Source: PLoS Genet. 2018 Oct 4;14(10):e1007651. doi: 10.1371/journal.pgen.1007651 (PMC6171792; doi:10.1371/journal.pgen.1007651)

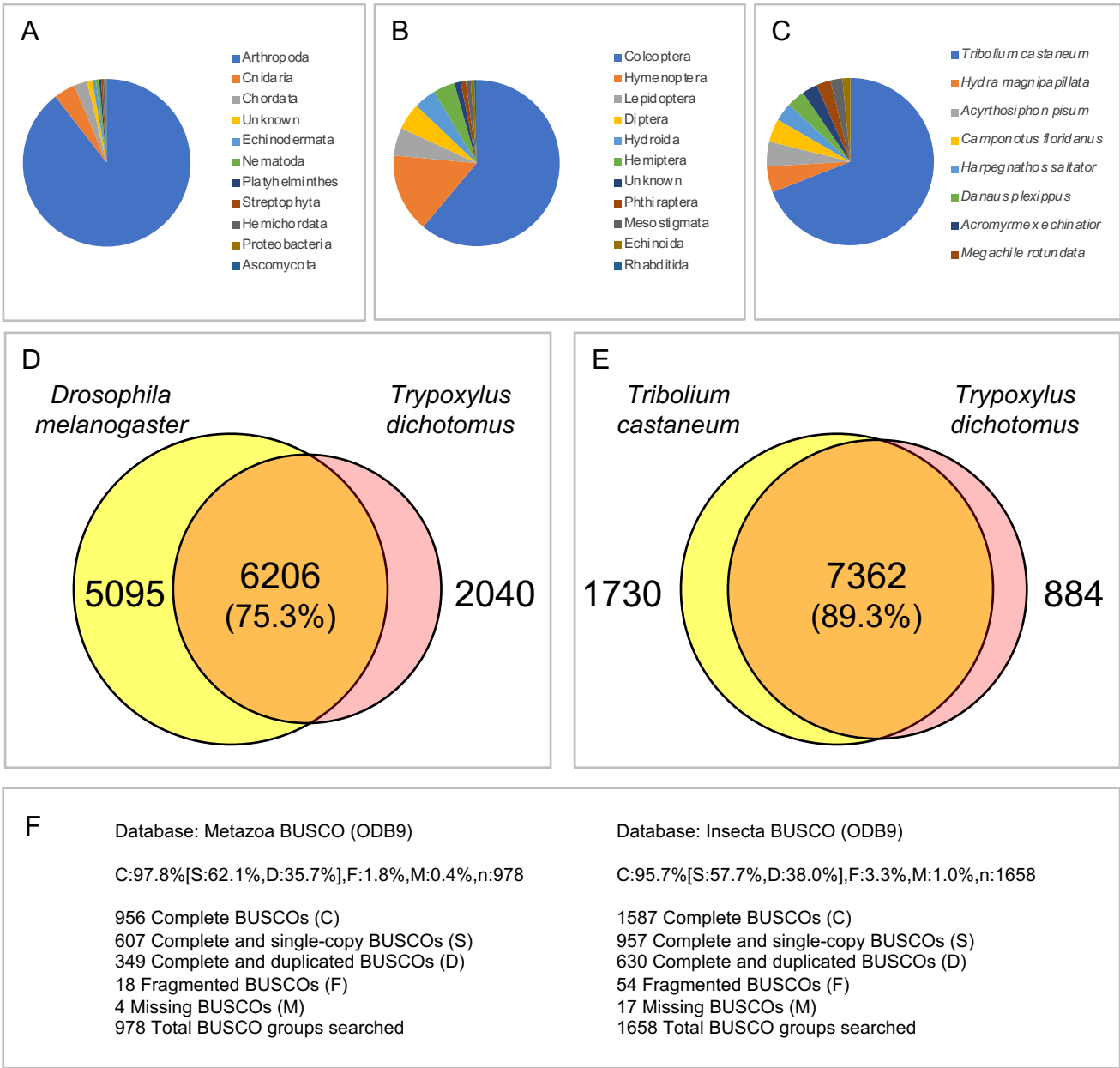

Supplement: S1 Fig — (A—C) Similarity search to NCBI nr database. The proportion of assembled transcripts that show the highest similarity to each group is indicated in phylum (A), order (B) and species (C) levels. (D and E) Similarity search to the fruit fly Drosophila melanogaster (D) and the red flour beetle Tribolium castaneum (E) sequences in OrthoDB5. The number of transcripts or genes belonging to each section and the percentage of T. dichotomus transcripts that have putative orthologous genes in OrthoDB5 database are indicated. (F) Results of BUSCO analysis against either Metazoa or Insecta database are shown. (PDF) [file pgen.1007651.s001.pdf]

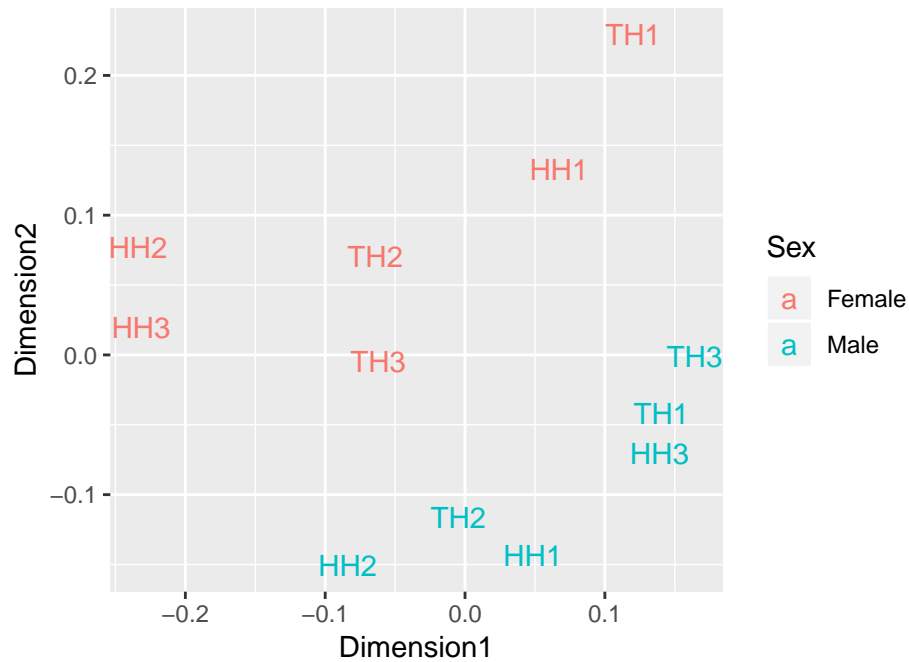

Supplement: S2 Fig — A multi-dimentional scaling plot of count data. (PDF) [file pgen.1007651.s002.pdf]

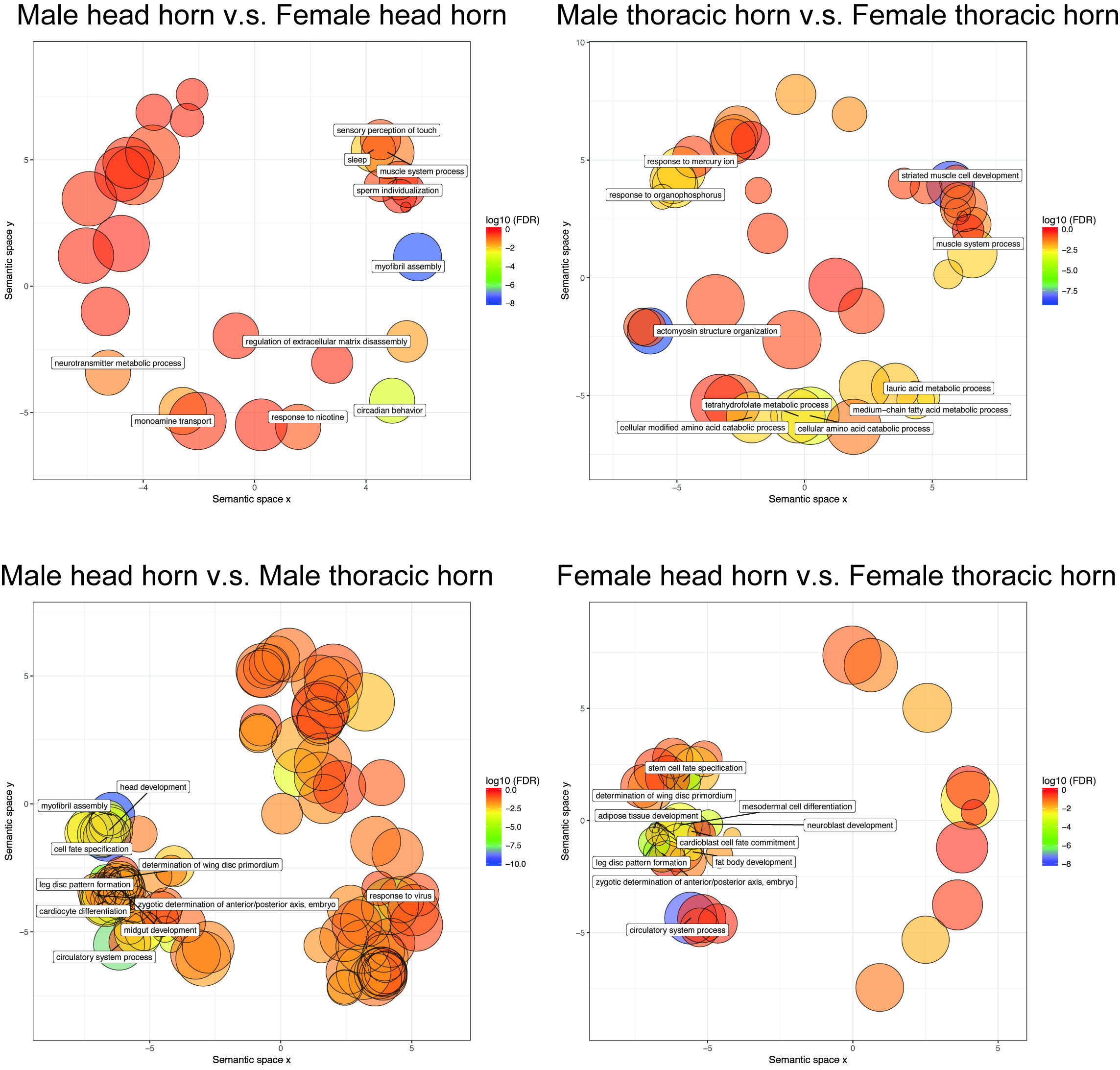

Supplement: S3 Fig — Enriched GO terms in each data set comparison that clustered based on semantic similarity of GO terms using REVIGO. The size of each point reflects the number of genes assigned to a GO term. Color indicates enrichment false discovery rate (FDR) in GO enrichment analysis using ErmineJ. GO terms enriched at FDR < 0.5 for each comparison in ErmineJ anlaysis are plotted. Descriptions of 10 GO terms that have the lowest FDR are shown on each plot. (JPG) [file pgen.1007651.s003.jpg]

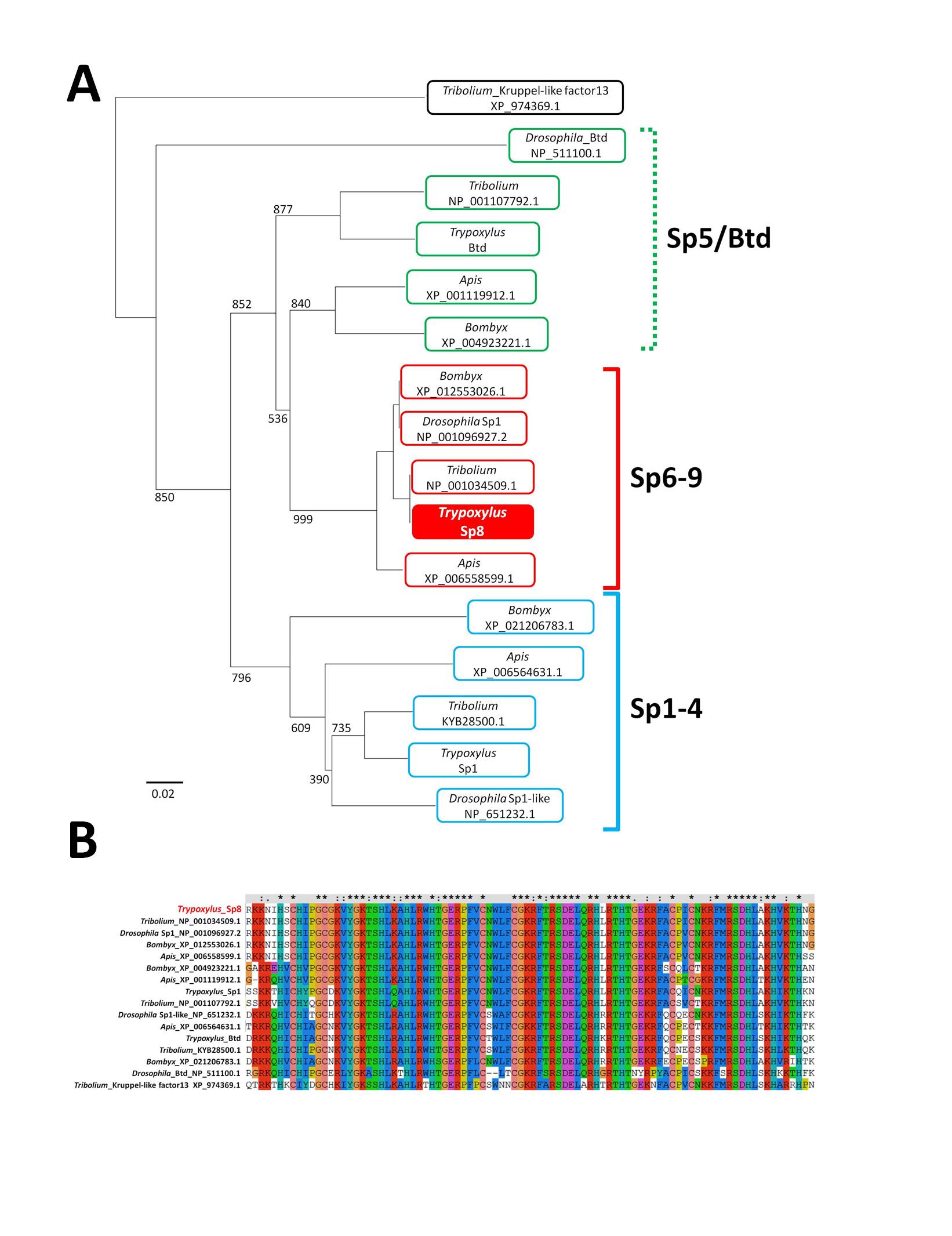

Supplement: S4 Fig — A phylogenetic tree (A) and multiple amino acid sequence alignment (B) of SP family genes in representative insects. The number at nodes indicate boot strap values in A. Amino acid identity and similarity among sequences are indicated by asterisks and dots, respectively. (JPG) [file pgen.1007651.s004.jpg]

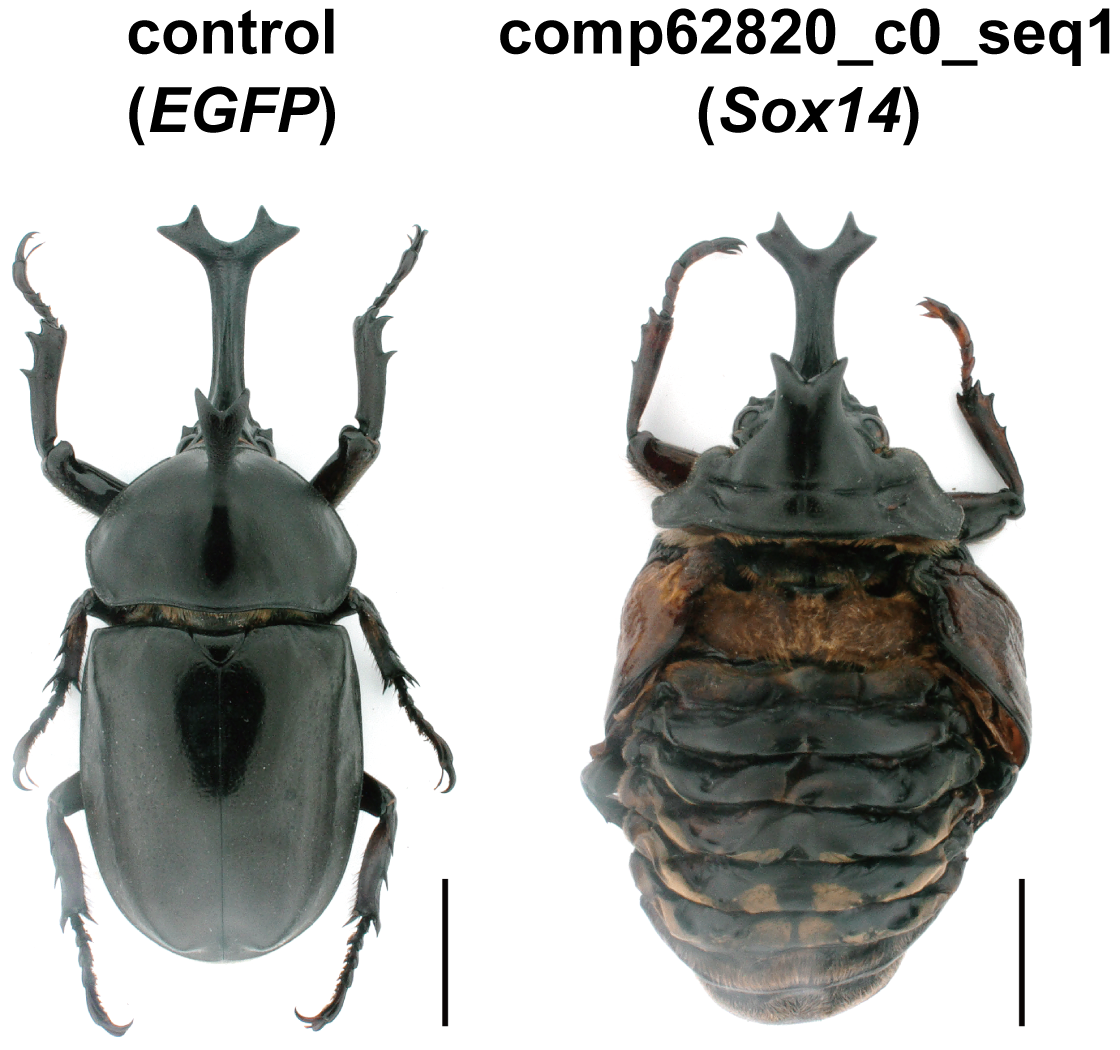

Supplement: S5 Fig — Dorsal view of adult male beetles. Scale bar is 1 cm. (TIF) [file pgen.1007651.s005.tif]

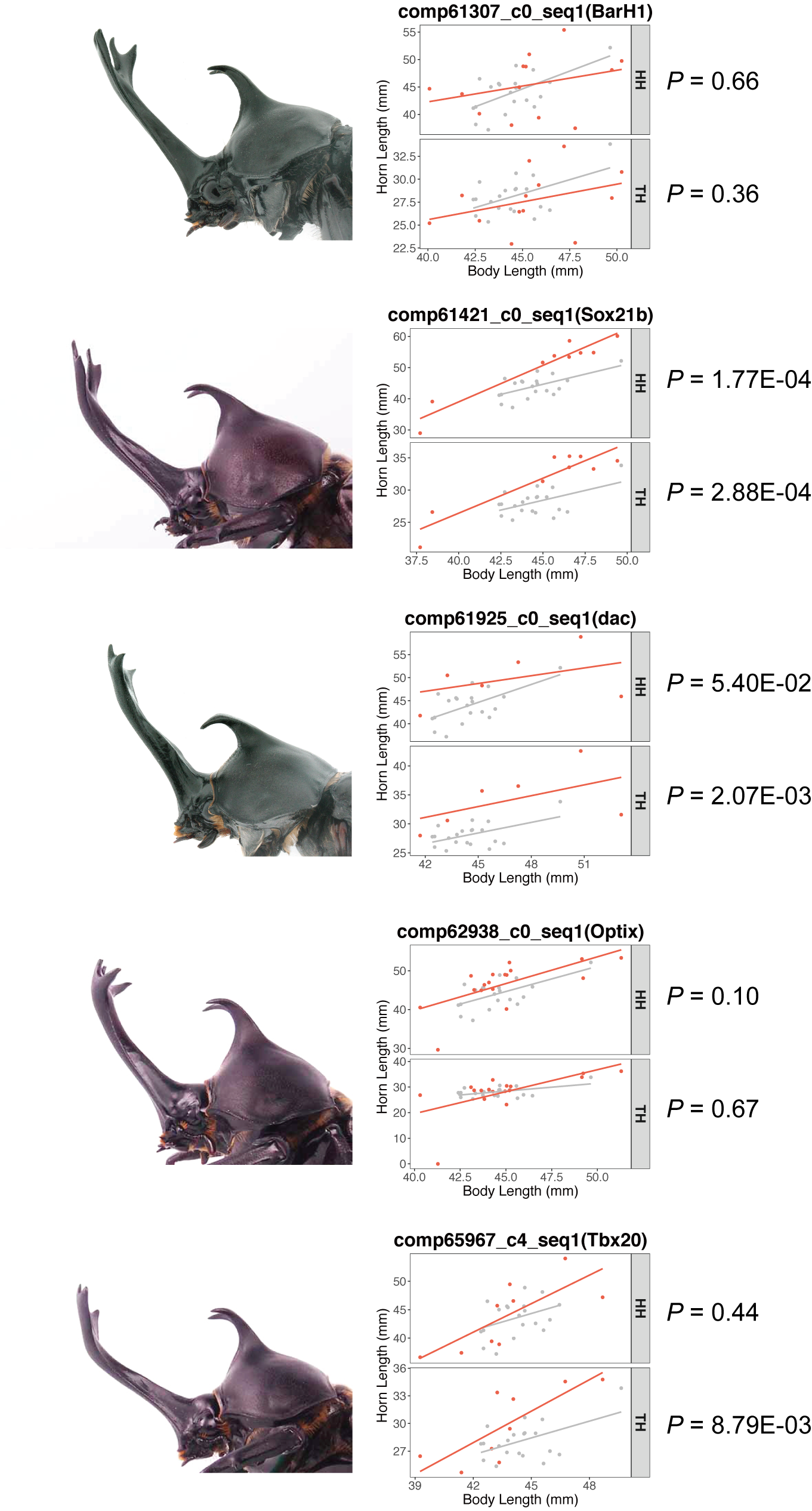

Supplement: S6 Fig — Lateral view of head and prothorax in RNAi beetles are displayed. Horn length and body length are plotted, and linear regression lines are drawn for each gene in red. Gray dots and gray regression lines are for EGFP RNAi beetles. P-values show significance of each RNAi treatment using Wald test on logistic linear regression. (TIF) [file pgen.1007651.s006.tif]

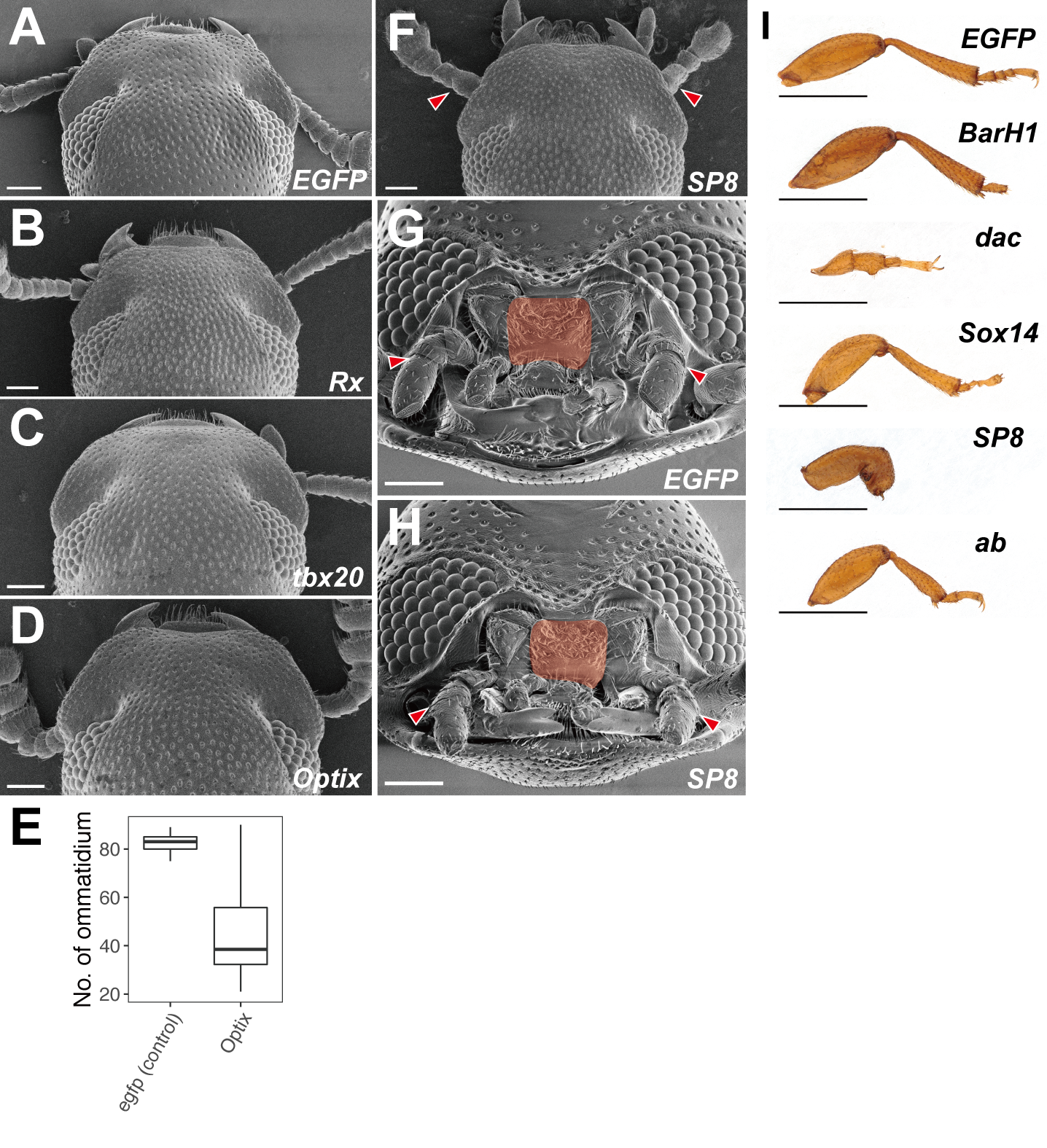

Supplement: S7 Fig — (A—D) Dorsal view of T. castaneum adult heads after RNAi treatments. (E) A box plot shows decreased number of ommatidium in Optix RNAi beetle compared to control. (F) Dorsal view of an SP8 RNAi beetle. Arrowheads in F indicate malformed antennae in SP8 RNAi beetles. (G and H) Frontal view of adult heads in EGFP (G) and SP8 (H) RNAi beetles. Arrowheads point to corresponding regions between G and H, which is fused by SP8 RNAi treatment (H). The dorsal part of the labrum is in red. (I) Adult metathoracic legs in T. castaneum after RNAi treatments. Scale bar is 0.1 mm in A—D, F—H, 0.5 mm in I. (TIF) [file pgen.1007651.s007.tif]

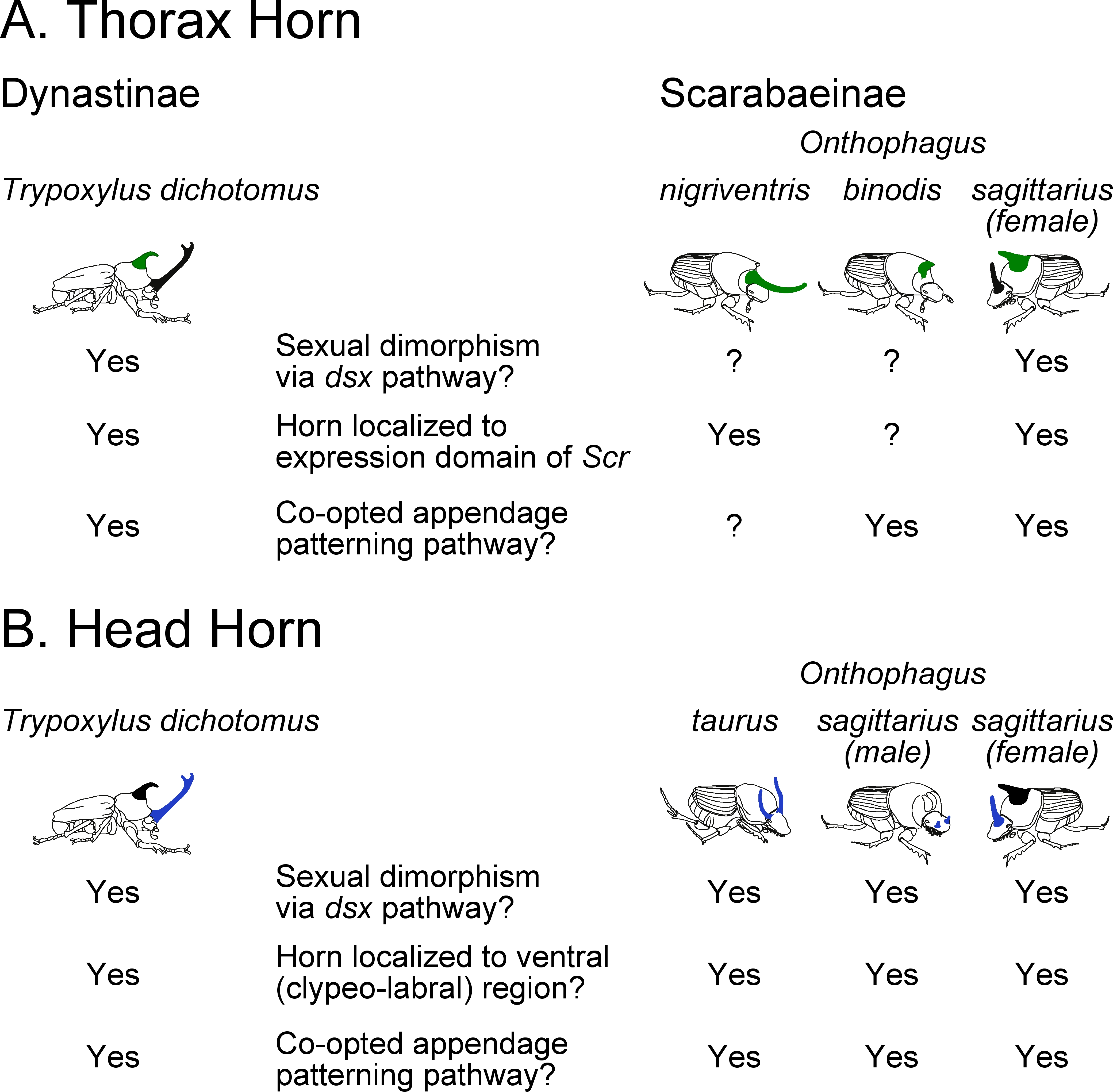

Supplement: S8 Fig — Both thoracic (A) and head (B) horns appear to have arisen through the repeated evolutionary co-option of parallel mechanistic processes, including the pathway regulating sexually dimorphic amounts of weapon growth (alternative splice forms of doublesex), the embryonic locations of horn outgrowth, and the co-option of portions of the insect appendage patterning pathway. However, the specific genes within the patterning pathway with the most pronounced effects on horn size differ somewhat between rhinoceros and dung beetle horns, consistent with their presumed independent evolutionary origins. Results summarized from [2], [5–6], [9–10], [21], [49]. (JPG) [file pgen.1007651.s008.jpg]
