# Supplementary material for: Rhinoceros beetle horn development reveals deep parallels with dung beetles
Source: PLoS Genet. 2018 Oct 4;14(10):e1007651. doi: 10.1371/journal.pgen.1007651 (PMC6171792; doi:10.1371/journal.pgen.1007651)
Supplement: S1 Table — (PDF) [file pgen.1007651.s009.pdf]

S1 Table. Summary of sequencing and de novo transcript assembly.

Total number of assembled reads	447,705,822
Number of trinity transcripts	127,986
Number of trinity genes	82,108
Number of identified genes*	8,246
Summary of trinity transcripts	
Total length (bp)	171,731,515
Average length (bp)	1,341.8
Median length (bp)	509
Max length (bp)	28,807
Min length (bp)	201
N50	3,158
GC content (%)	35.4

*Total number of trinity genes that hit a gene in OrthoDB5 at cutoff e-value 1e-4.
