# Supplementary material for: Rhinoceros beetle horn development reveals deep parallels with dung beetles
Source: PLoS Genet. 2018 Oct 4;14(10):e1007651. doi: 10.1371/journal.pgen.1007651 (PMC6171792; doi:10.1371/journal.pgen.1007651)
Supplement: S2 Table — (PDF) [file pgen.1007651.s010.pdf]

S2 Table. Number of differentially expressed genes with a cut off value of FDR < 0.05.

	Group1	Group2	High in Group1	High in Group2	Total
Intersexual	Male HH	Female HH	123	440	563
	Male TH	Female TH	99	77	176
Intrasexual	Male HH	Male TH	77	301	378
	Female HH	Female TH	321	115	436
