# Supplementary material for: Rhinoceros beetle horn development reveals deep parallels with dung beetles
Source: PLoS Genet. 2018 Oct 4;14(10):e1007651. doi: 10.1371/journal.pgen.1007651 (PMC6171792; doi:10.1371/journal.pgen.1007651)
Supplement: S3 Table — (PDF) [file pgen.1007651.s011.pdf]

S3 Table. Gene set analysis.

Male HH and female HH

ID	Definition	No. of genes	FDR
GO:0008010	structural constituent of chitin-based larval cuticle	95	1.81E-14
GO:0008011	structural constituent of pupal chitin-based cuticle	81	3.11E-14
GO:0008012	structural constituent of adult chitin-based cuticle	70	1.53E-13
GO:0030239	myofibril assembly	45	8.35E-09
GO:0008812	choline dehydrogenase activity	30	1.50E-07
GO:0042600	chorion	31	1.89E-07
GO:0031032	actomyosin structure organization	80	2.40E-07
GO:0030312	external encapsulating structure	49	2.15E-07
GO:0055001	muscle cell development	61	3.34E-07
GO:0055002	striated muscle cell development	61	3.34E-07
GO:0014866	skeletal myofibril assembly	12	2.75E-06
GO:0043292	contractile fiber	88	3.93E-05
GO:0044449	contractile fiber part	85	1.62E-04
GO:0050660	flavin adenine dinucleotide binding	66	4.59E-04
GO:0030016	myofibril	84	7.89E-04
GO:0048512	circadian behavior	99	7.62E-04
GO:0042752	regulation of circadian rhythm	59	9.36E-04
GO:0005859	muscle myosin complex	6	1.54E-03
GO:0030017	sarcomere	81	2.57E-03
GO:0030431	sleep	49	9.34E-03
GO:0016460	myosin II complex	9	0.01028323
GO:0036379	myofilament	10	0.01596015
GO:0008504	monoamine transmembrane transporter activity	5	0.02844772
GO:0010715	regulation of extracellular matrix disassembly	5	0.02721087
GO:0010716	negative regulation of extracellular matrix disassembly	5	0.02721087
GO:0015844	monoamine transport	5	0.02844772
GO:1903054	negative regulation of extracellular matrix organization	5	0.02721087
GO:0031011	Ino80 complex	13	0.04445588

GO:0097346	INO80-type complex	13	0.04445588
GO:0016490	structural constituent of peritrophic membrane	6	0.04902026
GO:1903053	regulation of extracellular matrix organization	6	0.04713487
GO:0042749	regulation of circadian sleep/wake cycle	36	0.04988948
GO:0045214	sarcomere organization	24	0.04966168
GO:0005328	neurotransmitter:sodium symporter activity	14	0.05036795
GO:0033202	DNA helicase complex	14	0.04868902
GO:0042133	neurotransmitter metabolic process	15	0.06283214
GO:0022410	circadian sleep/wake cycle process	39	0.06567686
GO:0003012	muscle system process	27	0.07466926

Male TH and female TH

ID	Name	No. of genes	FDR
GO:0031032	actomyosin structure organization	80	8.23E-10
GO:0030239	myofibril assembly	45	1.21E-09
GO:0055001	muscle cell development	61	2.00E-09
GO:0055002	striated muscle cell development	61	2.00E-09
GO:0043292	contractile fiber	88	1.39E-08
GO:0044449	contractile fiber part	85	1.36E-06
GO:0030016	myofibril	84	1.22E-05
GO:0030017	sarcomere	81	8.21E-05
GO:0014866	skeletal myofibril assembly	12	1.45E-04
GO:0005859	muscle myosin complex	6	1.87E-04
GO:0009986	cell surface	98	3.49E-04
GO:0009063	cellular amino acid catabolic process	44	6.69E-04
GO:0016460	myosin II complex	9	1.14E-03
GO:0036379	myofilament	10	1.74E-03
GO:0005416	cation:amino acid symporter activity	22	2.46E-03
GO:0016645	oxidoreductase activity, acting on the CH-NH group of donors	11	2.34E-03
GO:0045214	sarcomere organization	24	3.40E-03
GO:1901606	alpha-amino acid catabolic process	41	3.84E-03

GO:0016742	hydroxymethyl-, formyl- and related transferase activity	5	4.49E-03
GO:0042219	cellular modified amino acid catabolic process	5	4.25E-03
GO:0046653	tetrahydrofolate metabolic process	5	4.04E-03
GO:0016713	oxidoreductase activity, acting on paired donors, with incorporation or reduction of molecular oxygen, reduced iron-sulfur protein as one donor, and incorporation of one atom of oxygen	86	4.00E-03
GO:0018685	alkane 1-monooxygenase activity	86	4.00E-03
GO:0046683	response to organophosphorus	86	4.00E-03
GO:0046689	response to mercury ion	86	4.00E-03
GO:0048252	lauric acid metabolic process	86	4.00E-03
GO:0051791	medium-chain fatty acid metabolic process	86	4.00E-03
GO:0003012	muscle system process	27	4.54E-03
GO:0005280	hydrogen:amino acid symporter activity	14	4.48E-03
GO:0015495	gamma-aminobutyric acid:proton symporter activity	14	4.48E-03
GO:0006697	ecdysone biosynthetic process	93	6.18E-03
GO:0014074	response to purine-containing compound	93	5.94E-03
GO:0031000	response to caffeine	93	5.94E-03
GO:0016646	oxidoreductase activity, acting on the CH-NH group of donors, NAD or NADP as acceptor	6	6.15E-03
GO:0008205	ecdysone metabolic process	94	5.94E-03
GO:0016126	sterol biosynthetic process	94	5.72E-03
GO:0005884	actin filament	48	5.65E-03
GO:0045456	ecdysteroid biosynthetic process	95	5.76E-03
GO:0046680	response to DDT	95	5.58E-03
GO:0015185	gamma-aminobutyric acid transmembrane transporter activity	16	5.72E-03
GO:0015295	solute:proton symporter activity	16	5.55E-03
GO:0016054	organic acid catabolic process	74	7.12E-03
GO:0046395	carboxylic acid catabolic process	74	7.12E-03
GO:0016125	sterol metabolic process	100	7.13E-03

GO:0046165	alcohol biosynthetic process	100	6.93E-03
GO:0005044	scavenger receptor activity	18	8.14E-03
GO:0019010	farnesoic acid O-methyltransferase activity	20	0.01227635
GO:0005865	striated muscle thin filament	9	0.01668689
GO:0006936	muscle contraction	23	0.02061972
GO:0008171	O-methyltransferase activity	23	0.0201168
GO:0008168	methyltransferase activity	90	0.01981127
GO:0002118	aggressive behavior	42	0.02059928
GO:0002121	inter-male aggressive behavior	42	0.02059928
GO:0016741	transferase activity, transferring one-carbon groups	91	0.02024648
GO:0015026	coreceptor activity	10	0.02044482
GO:0035172	hemocyte proliferation	25	0.02505495
GO:0007352	zygotic specification of dorsal/ventral axis	11	0.02663528
GO:0016620	oxidoreductase activity, acting on the aldehyde or oxo group of donors, NAD or NADP as acceptor	47	0.03143295
GO:0016903	oxidoreductase activity, acting on the aldehyde or oxo group of donors	50	0.0411032
GO:0006760	folic acid-containing compound metabolic process	13	0.04250002
GO:0009620	response to fungus	52	0.04735228
GO:0050832	defense response to fungus	52	0.04735228
GO:0006730	one-carbon metabolic process	14	0.05147028
GO:0038024	cargo receptor activity	32	0.05722562
GO:0016459	myosin complex	33	0.06321335
GO:0042684	cardioblast cell fate commitment	16	0.07332017
GO:0035051	cardiocyte differentiation	35	0.07630014
GO:0007445	determination of imaginal disc primordium	17	0.08501713
GO:0001222	transcription corepressor binding	5	0.09851866
GO:0001226	RNA polymerase II transcription corepressor binding	5	0.09851866
GO:0004568	chitinase activity	5	0.09684886
GO:0035294	determination of wing disc primordium	5	0.09851866
GO:0048644	muscle organ morphogenesis	5	0.09523471
GO:0005121	Toll binding	18	0.09434306

GO:0019955	cytokine binding	18	0.0928214
GO:0043649	dicarboxylic acid catabolic process	18	0.09134804
GO:0051890	regulation of cardioblast differentiation	18	0.08992073
GO:2000736	regulation of stem cell differentiation	18	0.08992073
GO:0019732	antifungal humoral response	38	0.08966656
GO:0008028	monocarboxylic acid transmembrane transporter activity	39	0.09732605

Male HH and male TH

ID	Definition	No. of gene	FDR	
GO:0005550	pheromone binding	14	3.40E-08	H
GO:0030239	myofibril assembly	45	1.45E-07	
GO:0001708	cell fate specification	68	2.31E-07	
GO:0003705	RNA polymerase II distal enhancer sequence-specific DNA binding transcription factor activity	92	1.80E-06	
GO:0031032	actomyosin structure organization	80	1.56E-06	
GO:0055001	muscle cell development	61	2.96E-06	
GO:0055002	striated muscle cell development	61	2.96E-06	
GO:0014866	skeletal myofibril assembly	12	2.80E-06	
GO:0003013	circulatory system process	13	5.19E-06	
GO:0003015	heart process	13	5.19E-06	
GO:0007354	zygotic determination of anterior/posterior axis, embryo	54	4.63E-06	
GO:0007362	terminal region determination	45	5.80E-06	
GO:0043292	contractile fiber	88	2.16E-05	
GO:0042684	cardioblast cell fate commitment	16	2.15E-05	
GO:0005549	odorant binding	32	2.39E-05	
GO:0035223	leg disc pattern formation	25	3.79E-05	
GO:0007365	periodic partitioning	58	6.36E-05	
GO:0044449	contractile fiber part	85	7.01E-05	
GO:0009615	response to virus	48	7.74E-05	
GO:0010002	cardioblast differentiation	28	7.89E-05	
GO:0048665	neuron fate specification	29	1.01E-04	

GO:0060911	cardiac cell fate commitment	21	1.17E-04
GO:0035287	head segmentation	31	1.60E-04
GO:0001227	RNA polymerase II transcription regulatory region sequence-specific DNA binding transcription factor activity involved in negative regulation of transcription	32	1.99E-04
GO:0060322	head development	32	1.90E-04
GO:0044212	transcription regulatory region DNA binding	68	2.11E-04
GO:0030016	myofibril	84	2.68E-04
GO:0000975	regulatory region DNA binding	70	2.62E-04
GO:0001067	regulatory region nucleic acid binding	70	2.62E-04
GO:0001222	transcription corepressor binding	5	2.73E-04
GO:0001226	RNA polymerase II transcription corepressor binding	5	2.73E-04
GO:0035294	determination of wing disc primordium	5	2.73E-04
GO:0035051	cardiocyte differentiation	35	3.26E-04
GO:0007494	midgut development	36	3.95E-04
GO:0035050	embryonic heart tube development	28	6.75E-04
GO:0005859	muscle myosin complex	6	6.97E-04
GO:0030017	sarcomere	81	9.17E-04
GO:0000982	RNA polymerase II core promoter proximal region sequence-specific DNA binding transcription factor activity	68	1.10E-03
GO:0000976	transcription regulatory region sequence-specific DNA binding	42	1.14E-03
GO:0000977	RNA polymerase II regulatory region sequence-specific DNA binding	33	1.86E-03
GO:0014019	neuroblast development	23	2.01E-03
GO:0014018	neuroblast fate specification	8	2.61E-03
GO:0048866	stem cell fate specification	8	2.61E-03
GO:0002168	instar larval development	77	2.92E-03
GO:0048332	mesoderm morphogenesis	36	3.05E-03
GO:0001012	RNA polymerase II regulatory region DNA binding	37	3.58E-03
GO:0008061	chitin binding	37	3.49E-03
GO:0016460	myosin II complex	9	4.06E-03
GO:0001710	mesodermal cell fate commitment	17	4.40E-03

GO:0007445	determination of imaginal disc primordium	17	4.30E-03
GO:0048100	wing disc anterior/posterior pattern formation	17	4.21E-03
GO:0048333	mesodermal cell differentiation	17	4.40E-03
GO:0051890	regulation of cardioblast differentiation	18	5.57E-03
GO:0060795	cell fate commitment involved in formation of primary germ layer	18	5.46E-03
GO:2000736	regulation of stem cell differentiation	18	5.57E-03
GO:0001221	transcription cofactor binding	10	5.79E-03
GO:0001224	RNA polymerase II transcription cofactor binding	10	5.79E-03
GO:0007503	fat body development	10	5.67E-03
GO:0036379	myofilament	10	5.56E-03
GO:0060612	adipose tissue development	10	5.67E-03
GO:0007448	anterior/posterior pattern specification, imaginal disc	29	5.83E-03
GO:0008301	DNA binding, bending	19	6.55E-03
GO:0061448	connective tissue development	11	8.06E-03
GO:0042600	chorion	31	8.16E-03
GO:0001158	enhancer sequence-specific DNA binding	20	8.07E-03
GO:0035290	trunk segmentation	20	7.93E-03
GO:0035326	enhancer binding	20	8.07E-03
GO:0038024	cargo receptor activity	32	9.30E-03
GO:0001228	RNA polymerase II transcription regulatory region sequence-specific DNA binding transcription factor activity involved in positive regulation of transcription	46	0.0104316
GO:0007606	sensory perception of chemical stimulus	94	0.01037479
GO:0048066	developmental pigmentation	77	0.01032801
GO:0042689	regulation of crystal cell differentiation	12	0.01028756
GO:0001707	mesoderm formation	33	0.01021703
GO:0007218	neuropeptide signaling pathway	47	0.01102839
GO:0001704	formation of primary germ layer	34	0.01175038
GO:0031011	Ino80 complex	13	0.01365119
GO:0045611	negative regulation of hemocyte differentiation	13	0.01344436
GO:0097346	INO80-type complex	13	0.01365119
GO:0007367	segment polarity determination	35	0.01324423

GO:0043473	pigmentation	81	0.01341995
GO:0008015	blood circulation	6	0.01540626
GO:0048617	embryonic foregut morphogenesis	6	0.01518617
GO:0060047	heart contraction	6	0.01540626
GO:0035214	eye-antennal disc development	83	0.01545324
GO:0042659	regulation of cell fate specification	51	0.01620776
GO:0008363	larval chitin-based cuticle development	37	0.01664227
GO:0033202	DNA helicase complex	14	0.01643347
GO:0048073	regulation of eye pigmentation	14	0.01621436
GO:0048076	regulation of compound eye pigmentation	14	0.01621436
GO:0010453	regulation of cell fate commitment	52	0.0173415
GO:0042337	cuticle development involved in chitin-based cuticle molting cycle	38	0.01831858
GO:0035288	anterior head segmentation	15	0.02081239
GO:0097065	anterior head development	15	0.02081239
GO:0001103	RNA polymerase II repressing transcription factor binding	7	0.02307941
GO:0042690	negative regulation of crystal cell differentiation	7	0.02279091
GO:0030594	neurotransmitter receptor activity	72	0.02422843
GO:0048859	formation of anatomical boundary	56	0.02542537
GO:0045751	negative regulation of Toll signaling pathway	16	0.02552951
GO:0014016	neuroblast differentiation	57	0.02765538
GO:0007591	molting cycle, chitin-based cuticle	94	0.03230721
GO:0042303	molting cycle	94	0.03230721
GO:0001706	endoderm formation	8	0.03324698
GO:0004983	neuropeptide Y receptor activity	8	0.03286483
GO:0008010	structural constituent of chitin-based larval cuticle	95	0.03367694
GO:0001077	RNA polymerase II core promoter proximal region sequence-specific DNA binding transcription factor activity involved in positive regulation of transcription	44	0.03540805
GO:0008812	choline dehydrogenase activity	30	0.03636869
GO:0005044	scavenger receptor activity	18	0.03752186
GO:0005865	striated muscle thin filament	9	0.04569247
GO:0007510	cardioblast cell fate determination	9	0.04520115

GO:0046665	amnioserosa maintenance	9	0.04472029
GO:0060913	cardiac cell fate determination	9	0.04520115
GO:0004869	cysteine-type endopeptidase inhibitor activity	19	0.04457122
GO:0007380	specification of segmental identity, head	19	0.04410693
GO:0007442	hindgut morphogenesis	81	0.04416069
GO:0008011	structural constituent of pupal chitin-based cuticle	81	0.04371007
GO:0061525	hindgut development	81	0.04416069
GO:0008188	neuropeptide receptor activity	64	0.04699437
GO:0005884	actin filament	48	0.05007948
GO:0008528	G-protein coupled peptide receptor activity	65	0.05046019
GO:0007398	ectoderm development	33	0.05004062
GO:0007479	leg disc proximal/distal pattern formation	20	0.0503094
GO:0042688	crystal cell differentiation	20	0.04982566
GO:0060538	skeletal muscle organ development	84	0.05159818
GO:0030312	external encapsulating structure	49	0.05264388
GO:0007492	endoderm development	10	0.05501141
GO:0008407	chaeta morphogenesis	67	0.05632248
GO:0042562	hormone binding	21	0.05749591
GO:0048526	imaginal disc-derived wing expansion	21	0.05697322
GO:0007402	ganglion mother cell fate determination	22	0.0675625
GO:0035289	posterior head segmentation	22	0.06695927
GO:0060573	cell fate specification involved in pattern specification	11	0.07020606
GO:0048546	digestive tract morphogenesis	90	0.07359719
GO:0048568	embryonic organ development	71	0.07385299
GO:0008171	O-methyltransferase activity	23	0.07662465
GO:0045861	negative regulation of proteolysis	23	0.07596974
GO:0001653	peptide receptor activity	72	0.07793549
GO:0008354	germ cell migration	73	0.08355753
GO:0007516	hemocyte development	12	0.08640444
GO:0007449	proximal/distal pattern formation, imaginal disc	24	0.08630867
GO:0045214	sarcomere organization	24	0.08560122
GO:0006030	chitin metabolic process	75	0.09410701

GO:0006726	eye pigment biosynthetic process	40	0.09792874
GO:0042441	eye pigment metabolic process	40	0.09792874
GO:0043324	pigment metabolic process involved in developmental pigmentation	40	0.09792874
GO:0043474	pigment metabolic process involved in pigmentation	40	0.09792874
GO:0004867	serine-type endopeptidase inhibitor activity	25	0.09738478

Female HH and female TH

ID	Definition	No. of genes	FDR
GO:0008011	structural constituent of pupal chitin-based cuticle	81	1.17E-17
GO:0008010	structural constituent of chitin-based larval cuticle	95	2.18E-17
GO:0008012	structural constituent of adult chitin-based cuticle	70	9.55E-16
GO:0042600	chorion	31	6.15E-13
GO:0008812	choline dehydrogenase activity	30	1.07E-11
GO:0030312	external encapsulating structure	49	3.62E-11
GO:0042684	cardioblast cell fate commitment	16	4.50E-09
GO:0003013	circulatory system process	13	2.16E-08
GO:0003015	heart process	13	2.16E-08
GO:0003705	RNA polymerase II distal enhancer sequence-specific DNA binding transcription factor activity	92	4.39E-08
GO:0001708	cell fate specification	68	5.55E-08
GO:0035050	embryonic heart tube development	28	5.63E-08
GO:0060911	cardiac cell fate commitment	21	6.12E-08
GO:0050660	flavin adenine dinucleotide binding	66	3.47E-07
GO:0035051	cardiocyte differentiation	35	5.40E-07
GO:0001222	transcription corepressor binding	5	9.87E-07
GO:0001226	RNA polymerase II transcription corepressor binding	5	9.87E-07
GO:0035294	determination of wing disc primordium	5	9.87E-07
GO:0010002	cardioblast differentiation	28	9.43E-07
GO:0005550	pheromone binding	14	1.22E-06
GO:0007354	zygotic determination of anterior/posterior axis, embryo	54	3.26E-06
GO:0044212	transcription regulatory region DNA binding	68	3.82E-06
GO:0000975	regulatory region DNA binding	70	5.10E-06

GO:0001067	regulatory region nucleic acid binding	70	5.10E-06
GO:0000976	transcription regulatory region sequence-specific DNA binding	42	3.54E-05
GO:0014018	neuroblast fate specification	8	3.57E-05
GO:0048866	stem cell fate specification	8	3.57E-05
GO:0001227	RNA polymerase II transcription regulatory region sequence-specific DNA binding transcription factor activity involved in negative regulation of transcription	32	4.27E-05
GO:0005549	odorant binding	32	4.09E-05
GO:0000977	RNA polymerase II regulatory region sequence-specific DNA binding	33	5.09E-05
GO:0007362	terminal region determination	45	5.37E-05
GO:0035223	leg disc pattern formation	25	8.73E-05
GO:0007445	determination of imaginal disc primordium	17	1.15E-04
GO:0048100	wing disc anterior/posterior pattern formation	17	1.11E-04
GO:0001012	RNA polymerase II regulatory region DNA binding	37	1.09E-04
GO:0001221	transcription cofactor binding	10	1.10E-04
GO:0001224	RNA polymerase II transcription cofactor binding	10	1.10E-04
GO:0042659	regulation of cell fate specification	51	1.33E-04
GO:0051890	regulation of cardioblast differentiation	18	1.44E-04
GO:2000736	regulation of stem cell differentiation	18	1.44E-04
GO:0010453	regulation of cell fate commitment	52	1.49E-04
GO:0048665	neuron fate specification	29	2.03E-04
GO:0048568	embryonic organ development	71	2.63E-04
GO:0001158	enhancer sequence-specific DNA binding	20	2.59E-04
GO:0035326	enhancer binding	20	2.59E-04
GO:0007365	periodic partitioning	58	3.44E-04
GO:0014019	neuroblast development	23	6.04E-04
GO:0008061	chitin binding	37	1.01E-03
GO:0001710	mesodermal cell fate commitment	17	1.80E-03
GO:0048333	mesodermal cell differentiation	17	1.80E-03
GO:0014016	neuroblast differentiation	57	2.28E-03
GO:0007448	anterior/posterior pattern specification, imaginal disc	29	2.31E-03

GO:0060795	cell fate commitment involved in formation of primary germ layer	18	2.28E-03
GO:0014017	neuroblast fate commitment	43	2.50E-03
GO:0048867	stem cell fate determination	43	2.45E-03
GO:0007606	sensory perception of chemical stimulus	94	2.54E-03
GO:0007503	fat body development	10	2.68E-03
GO:0060612	adipose tissue development	10	2.68E-03
GO:0008301	DNA binding, bending	19	2.72E-03
GO:0035287	head segmentation	31	2.96E-03
GO:0060322	head development	32	3.51E-03
GO:0061448	connective tissue development	11	3.82E-03
GO:0009615	response to virus	48	4.42E-03
GO:0007402	ganglion mother cell fate determination	22	5.30E-03
GO:0007367	segment polarity determination	35	5.52E-03
GO:0048865	stem cell fate commitment	50	5.47E-03
GO:0000982	RNA polymerase II core promoter proximal region sequence-specific DNA binding transcription factor activity	68	6.11E-03
GO:0007494	midgut development	36	6.16E-03
GO:0008015	blood circulation	6	0.01006664
GO:0060047	heart contraction	6	0.01006664
GO:0048066	developmental pigmentation	77	0.01392974
GO:0042752	regulation of circadian rhythm	59	0.01448678
GO:0070491	repressing transcription factor binding	16	0.01621211
GO:0001103	RNA polymerase II repressing transcription factor binding	7	0.01622885
GO:0035310	notum cell fate specification	7	0.01597528
GO:0042690	negative regulation of crystal cell differentiation	7	0.0157295
GO:0043473	pigmentation	81	0.01794804
GO:0001706	endoderm formation	8	0.02401788
GO:0035309	wing and notum subfield formation	18	0.02400325
GO:0007380	specification of segmental identity, head	19	0.02944754
GO:0001707	mesoderm formation	33	0.03024848
GO:0007510	cardioblast cell fate determination	9	0.03344346

GO:0046665	amnioserosa maintenance	9	0.03297896
GO:0060913	cardiac cell fate determination	9	0.03344346
GO:0001704	formation of primary germ layer	34	0.03340143
GO:0035290	trunk segmentation	20	0.03373173
GO:0048332	mesoderm morphogenesis	36	0.04249492
GO:0007492	endoderm development	10	0.04390704
GO:0006030	chitin metabolic process	75	0.04954876
GO:0007379	segment specification	38	0.05248848
GO:0002168	instar larval development	77	0.05637707
GO:0060573	cell fate specification involved in pattern specification	11	0.05642147
GO:0031032	actomyosin structure organization	80	0.0686965
GO:0035265	organ growth	41	0.0706468
GO:0007516	hemocyte development	12	0.07133237
GO:0042689	regulation of crystal cell differentiation	12	0.07048318
GO:0035214	eye-antennal disc development	83	0.0809256
GO:0042693	muscle cell fate commitment	13	0.08804586
GO:0045611	negative regulation of hemocyte differentiation	13	0.08703384
GO:0001077	RNA polymerase II core promoter proximal region sequence-specific DNA binding transcription factor activity involved in positive regulation of transcription	44	0.09042114
