# Supplementary material for: Rhinoceros beetle horn development reveals deep parallels with dung beetles
Source: PLoS Genet. 2018 Oct 4;14(10):e1007651. doi: 10.1371/journal.pgen.1007651 (PMC6171792; doi:10.1371/journal.pgen.1007651)
Supplement: S4 Table — (PDF) [file pgen.1007651.s012.pdf]

S4 Table. Summary of RNAi analysis.

ID[*1]	Description	Type[*2]	Comparison[*3]				Sex	Injected RNA (μg)	N	Eclosed to adult
			Male HH vs Female HH	Male TH vs Female TH	Male HH vs Male TH	Female HH vs Female TH				
-	EGFP	-					Male	10	42	27
							Female	10	17	11
comp3738_c0	Wnt-10b	SM			Male TH		Male	1	2	2
								5	2	2
								10	2	2
							Female	1	2	2
								5	2	2
								10	2	2
comp40568_c0	Wnt6 protein precursor	SM			Male TH		Male	1	2	2
								5	2	2
								10	2	2
							Female	1	2	2
								5	2	2
								10	2	2
comp45679_c0	Retinal homeobox protein	TF			Male HH	Female HH	Male	10	2	2
								20	19	12
								26.5	2	2
							Female	10	2	2
								20	10	7
comp47535_c0	T-box protein Tbx1 >optomotor blind related gene 1 protein	TF		Female TH		Female TH	Male	7.4	2	1
							Female	7.4	2	2
comp49439_c0	Wnt7-1	SM	Male HH	Male TH			Male	1	2	2
								5	18	8
								10	22	8
							Female	5	10	0
								10	10	2
comp49933_c0	bone morphogenetic protein 2-like	SM				Female HH	Male	10	2	2
							Female	10	2	2
comp52023_c0	N、Calcium-binding EGF-like domain	SM	Male HH				Male	10	2	2
							Female	10	2	2
comp52609_c0	hormone receptor 83	SM			Male TH		Male	3	2	2
								8.4	2	0
							Female	3	2	2
								8.4	2	0
comp56528_c0	muscle LIM protein isoform 1	TF	Female HH	Female TH	Male TH		Male	5.8	2	2
							Female	5.8	2	2
comp56805_c1	cycle	TF	Female HH				Male	10	2	2
							Female	10	2	2
comp56808_c0	smad nuclear interacting protein	TF	Female HH			Female HH	Male	10	2	2
							Female	10	2	2
comp56914_c0	brinker	TF	Male HH	Male TH			Male	9.8	2	2
							Female	9.8	2	2
comp57995_c0	Pox neuro	TF		Male TH		Female HH	Male	7.5	2	2
							Female	7.5	2	2
comp58454_c0	sloppy paired 2	TF			Male HH	Female HH	Male	10	2	2
								20	4	4
							Female	10	2	2
								20	2	2
comp58528_c1	odd-skipped	TF				Female TH	Male	7.2	2	2
							Female	7.2	2	2
comp59045_c0	teashirt-like protein	TF			Male TH	Female TH	Male	8.7	2	2
							Female	8.7	2	2
comp59576_c0	Zinc finger, C3HC4 type (RING finger)	TF	Female HH		Male TH		Male	10	2	2
							Female	10	2	2
comp59701_c0	vestigial	TF			Male TH	Female TH	Male	5.5	2	2
								10	2	2
								20	2	2
							Female	5.5	2	2
								10	2	1
								20	2	1
comp59895_c0	Antennapedia	TF			Male TH	Female TH	Male	10	2	1
							Female	10	2	1
comp60275_c0	grain (dGATAc)	TF			Male TH	Female TH	Male	10	2	0
							Female	10	2	0
comp61134_c0	zinc finger MYM-type protein 1-like	TF				Female HH	Male	10	2	2
							Female	10	2	2
comp61267_c0	escargot	TF			Male TH		Male	10	2	2
							Female	10	3	3
comp61307_c0	BarH1	TF	Male HH				Male	10	20	15
								20	4	2
								30	4	3
							Female	10	11	11
comp61360_c0	TGF-beta family, myostatin>myoglianin	SM			Male TH	Female TH	Male	10	2	2
							Female	10	2	2
comp61421_c0	Sox21b	TF			Male HH	Female HH	Male	10	2	2
								30	9	9
							Female	10	2	2
								30	9	8
comp61853_c0	epidermal growth factor-like protein	SM	Female HH				Male	10	2	2
							Female	10	2	2
comp61915_c0	Lyra (senseless)	TF					Male	10	2	2
							Female	10	2	2
comp61925_c0	dachshund	TF			Male HH	Female HH	Male	10	7	6
								20	2	1
								30	2	2
							Female	10	18	9

comp62113_c0	Tnebrio hormone receptor 4	SM	Female HH			Female HH	Male	5	2	0
								9.2	2	0
							Female	5	2	0
								9.2	2	0
comp62820_c0	SOX-14	TF			Male TH		Male	2.5	13	3
								10	26	8
								20	2	1
							Female	2.5	3	1
								10	17	3
								20	3	2
comp62938_c0	Optix	TF			Male HH	Female HH	Male	10	23	16
								30	9	5
							Female	10	11	4
								30	3	3
comp63178_c0	Sp-like zinc finger transcription factor (Sp8)	TF			Male HH	Female HH	Male	1	10	9
								10	10	10
								20	3	0
								40	3	0
							Female	1	5	5
								10	8	8
comp63338_c0	orthodenticle-2	TF		Male TH	Male HH	Female HH	Male	10	5	4
								20	26	16
								25	5	4
								50	2	0
								100	2	0
							Female	10	5	4
								20	5	5
								25	5	5
								50	2	2
								100	3	1
comp63540_c0	epidermal growth factor-like protein	SM			Male TH		Male	10	2	2
								20	2	2
							Female	10	2	1
								20	2	2
comp63721_c0	abrupt	TF			Male TH	Female TH	Male	6	2	2
								20	9	6
								23.4	2	2
							Female	10	12	7
comp63774_c2	disco-related	TF			Male HH		Male	8	2	2
								18.5	2	1
								20.5	3	3
							Female	8	2	2
								18.5	2	1
								20.5	1	1
comp64312_c0	BMP and activin membrane-bound inhibitor-like	SM			Male TH		Male	10	2	2
							Female	10	2	2
comp64379_c1	engrailed	TF				Female TH	Male	1	2	2
								10	2	2
							Female	1	2	2
								10	2	2
comp65258_c1	drumstick	TF	Female HH		Male TH		Male	10	2	2
							Female	10	2	2
comp65763_c0	iroquois-class homeodomain protein	TF				Female TH	Male	6.3	2	1
								20	2	2
							Female	6.3	2	2
								20	2	0
comp65832_c1	Winged helix-turn helix, Homeodomain-like domain	TF			Male TH		Male	5.5	2	2
							Female	5.5	2	2
comp65846_c0	eyegone	TF	Female HH		Male TH		Male	7.7	2	2
								20	10	5
								88	2	0
							Female	7.7	2	2
								20	3	2
								88	2	2
comp65931_c0	optomotor-blind-like	TF			Male TH	Female TH	Male	7.7	2	1
								15	3	3
							Female	7.7	2	0
								15	2	1
comp65967_c4	T-box transcription factor 20	TF	Male HH		Male HH	Female HH	Male	8.8	2	2
								20	10	9
								20.5	4	3
							Female	8.8	2	2
								10	9	6
comp66046_c4	spalt	TF				Female TH	Male	10	2	2
							Female	10	2	2
comp66333_c0	Sex combs reduced	TF			Male TH	Female TH	Male	10	11	11
								20	3	2
								40	2	1
							Female	10	7	7
								20	2	2
comp66406_c1	pannier	TF			Male TH		Male	10	23	8
							Female	10	9	6
comp102925_c0	brachyenteron (Brachyury (T) homolog)	TF			Male TH		Male	10	2	2
							Female	10	2	2
comp786117_c0	Transcription factor 21 (Podocyte-expressed 1) (Pod-1) (Epicardin) (Capsulin)	TF	Female HH			Female HH	Male	8.8	2	2
							Female	8.8	2	2

*1 Blue: some visible change on horn phenotype was detected in initial screening, but no significant change in both shape and size.
Orange: statistically significant change was detected for shape and/or size.
*2 SM, signaling molecule; TF, transcription factor
*3 Group that shows higher expression in the comparison (FDR < 0.05)
