# Supplementary material for: Rhinoceros beetle horn development reveals deep parallels with dung beetles
Source: PLoS Genet. 2018 Oct 4;14(10):e1007651. doi: 10.1371/journal.pgen.1007651 (PMC6171792; doi:10.1371/journal.pgen.1007651)
Supplement: S5 Table — (PDF) [file pgen.1007651.s013.pdf]

S5 Table. Wald test for RNAi effects on shape.

HH central groove

Target gene	Body length				dsRNA treatment			
	Estimate	Standard error	*t*	*P*	Estimate	Standard error	*t*	*P*
comp45679_c0_seq1 (Rx)	0.3651	0.2698	1.353	0.188	12.9706	1.506	8.613	4.31E-09 ***
comp49439_c0_seq2 (Wnt7-1)	0.7564	0.3226	2.345	2.70E-02 *	-2.7194	2.0571	-1.322	0.198
comp61307_c0_seq1 (BarH1)	0.9626	0.3408	2.824	0.00835 **	-0.587	1.5505	-0.379	0.70768
comp61421_c0_seq1 (Sox21b)	1.6501	0.3322	4.967	3.67E-05 ***	-6.4201	1.8449	-3.48	0.00178 **
comp61925_c0_seq1 (dac)	-0.09834	0.33471	-0.294	0.77155	-6.23479	2.08116	-2.996	0.00645 **
comp62820_c0_seq1 (Sox14)	-0.06446	0.3223	-0.2	0.843306	7.22106	1.83656	3.932	7.12E-04 ***
comp62938_c0_seq1 (Optix)	0.1291	0.2212	0.584	5.63E-01	13.8821	0.9756	14.229	7.02E-16 ***
comp63178_c0_seq2 (SP8)	1.6913	0.4061	4.164	3.04E-04 ***	-5.4664	2.6962	-2.027	5.30E-02 .
comp63338_c0_seq1 (otd2)	0.1264	0.4337	0.292	0.772	-0.7681	1.8937	-0.406	0.687
comp63721_c0_seq1 (ab)	0.3923	0.3501	1.12	2.74E-01	8.8896	1.6505	5.386	0.000018 ***
comp65846_c0_seq1 (eyg)	0.4314	0.3561	1.211	0.235	-1.3315	1.5732	-0.846	0.404
comp65967_c4_seq1 (Tbx20)	0.7807	0.4162	1.876	0.072 .	-3.1495	1.8204	-1.73	0.0955 .
comp66333_c0_seq1 (Scr)	0.4237	0.4449	0.953	0.34927	-7.8398	2.2385	-3.502	0.00162 **
comp66406_c1_seq1 (pnr)	0.03621	0.31298	0.116	0.90891	-7.51332	2.13212	-3.524	0.00182 **

HH side grooves

Target gene	Body size				dsRNA treatment			
	Estimate	Standard error	*t*	*P*	Estimate	Standard error	*t*	*P*
comp45679_c0_seq1 (Rx)	0.21745	0.07697	2.825	8.96E-03 **	1.84138	0.42972	4.285	2.22E-04 ***
comp49439_c0_seq2 (Wnt7-1)	0.004723	0.081186	0.058	0.954	-0.460449	0.517663	-0.889	0.382
comp61307_c0_seq1 (BarH1)	0.19168	0.07836	2.45E+00	2.05E-02 *	0.79242	0.35648	2.22E+00	0.0339 *
comp61421_c0_seq1 (Sox21b)	0.133	0.0582	2.29E+00	3.07E-02 *	3.2604	0.3232	1.01E+01	1.77E-10 ***
comp61925_c0_seq1 (dac)	-0.01168	0.07529	-0.155	0.87802	2.03244	0.46812	4.342	0.00024 ***
comp62820_c0_seq1 (Sox14)	0.02166	0.08256	0.262	0.795	2.60855	0.47043	5.55E+00	1.42E-05 ***
comp62938_c0_seq1 (Optix)	0.11013	0.09199	1.197	0.24	0.32696	0.40581	0.806	0.426
comp63178_c0_seq2 (SP8)	0.51074	0.09252	5.52E+00	0.00000856 ***	-1.82847	0.6142	-2.98E+00	6.22E-03 **
comp63338_c0_seq1 (otd2)	-0.06119	0.12212	-0.501	0.619	-0.13264	0.5333	-0.249	0.805
comp63721_c0_seq1 (ab)	0.13712	0.08167	1.679	0.107	3.57026	0.38498	9.274	3.11E-09 ***
comp65846_c0_seq1 (eyg)	0.2845	0.1282	2.219	0.034 *	-0.6135	0.5664	-1.083	0.287
comp65967_c4_seq1 (Tbx20)	0.08603	0.09065	0.949	0.351	2.14658	0.39654	5.413	0.0000113 ***
comp66333_c0_seq1 (Scr)	-0.06362	0.12412	-0.513	0.612433	-2.62595	0.62457	-4.204	2.57E-04 ***
comp66406_c1_seq1 (pnr)	0.05663	0.07136	0.794	0.436	-0.32352	0.48615	-0.665	0.512

TH groove

Target gene	Body size				dsRNA treatment			
	Estimate	Standard error	*t*	*P*	Estimate	Standard error	*t*	*P*
comp45679_c0_seq1 (Rx)	0.18696	0.07499	2.493	1.88E-02 *	-1.86064	0.49912	-3.728	8.67E-04 ***
comp49439_c0_seq2 (Wnt7-1)	0.10671	0.06453	1.654	0.11	-0.44026	0.50383	-0.874	0.39
comp61307_c0_seq1 (BarH1)	0.04182	0.06347	6.59E-01	5.15E-01	0.32522	0.35455	9.17E-01	0.366
comp61421_c0_seq1 (Sox21b)	0.2339	0.072	3.25E+00	3.02E-03 **	-2.1553	0.4807	-4.48E+00	0.000114 ***
comp61925_c0_seq1 (dac)	0.07501	0.06059	1.238	0.2272	-0.94437	0.45779	-2.063	0.0497 *
comp62820_c0_seq1 (Sox14)	0.11362	0.07478	1.519	0.142	-0.52753	0.58749	-8.98E-01	3.79E-01
comp62938_c0_seq1 (Optix)	0.23636	0.08507	2.778	0.008625 **	-1.84523	0.45301	-4.073	0.000243 ***
comp63178_c0_seq2 (SP8)	0.2506	0.0974	2.57E+00	0.015901 *	-3.2869	0.7436	-4.42E+00	1.44E-04 ***
comp63338_c0_seq1 (otd2)	0.16927	0.06698	2.527	0.0159 *	-0.40165	0.34998	-1.148	0.2585
comp63721_c0_seq1 (ab)	0.09106	0.06776	1.344	0.192	2.32698	0.46891	4.963	4.57E-05 ***
comp65846_c0_seq1 (eyg)	0.19835	0.06557	3.025	0.00479 **	-0.18824	0.35589	-0.529	0.60039
comp65967_c4_seq1 (Tbx20)	0.21865	0.08867	2.466	0.02005 *	-1.87827	0.51442	-3.651	0.00106 **
comp66333_c0_seq1 (Scr)	0.07092	0.07262	0.977	0.3368	0.79636	0.44468	1.791	8.38E-02 .
comp66406_c1_seq1 (pnr)	0.03363	0.04807	0.699	0.49	3.28979	0.38282	8.593	3.3E-09 ***
