# Supplementary material for: Rhinoceros beetle horn development reveals deep parallels with dung beetles
Source: PLoS Genet. 2018 Oct 4;14(10):e1007651. doi: 10.1371/journal.pgen.1007651 (PMC6171792; doi:10.1371/journal.pgen.1007651)
Supplement: S6 Table — (PDF) [file pgen.1007651.s014.pdf]

S6 Table. Wald test for RNAi effec horn length.

Head horn length

Target gene	Body length				dsRNA treatment			
	Estimate	Standard error	*t*	*P*	Estimate	Standard error	*t*	*P*
comp45679_c0_seq1 (Rx)	0.8688	0.336	2.586	0.0157 *	9.3529	1.8759	4.986	3.49E-05 ***
comp49439_c0_seq2 (Wnt7-1)	1.4839	0.2859	5.19	2.04E-05 ***	0.8185	1.8231	0.449	0.657
comp61307_c0_seq1 (BarH1)	0.829	0.328	2.527	0.017 *	-0.6588	1.4921	-0.441	0.662
comp61421_c0_seq1 (Sox21b)	2.0378	0.2331	8.743	3.21E-09 ***	-5.6549	1.2942	-4.369	0.000177 ***
comp61925_c0_seq1 (dac)	0.8276	0.3039	2.723	0.0121 *	-3.8385	1.8899	-2.031	0.054
comp62820_c0_seq1 (Sox14)	0.8231	0.2735	3.009	0.00646 **	15.0477	1.5587	9.654	2.28E-09 ***
comp62938_c0_seq1 (Optix)	1.3529	0.2735	4.947	2.01E-05 ***	-2.0145	1.2064	-1.67	0.104
comp63178_c0_seq2 (SP8)	1.4484	0.2882	5.027	2.84E-05 ***	-7.9247	1.8564	-4.269	2.17E-04 ***
comp63338_c0_seq1 (otd2)	0.4754	0.4556	1.044	0.304	0.9598	1.9896	0.482	0.633
comp63721_c0_seq1 (ab)	1.6076	0.2811	5.719	7.98E-06 ***	5.795	1.325	4.374	0.000222 ***
comp65846_c0_seq1 (eyg)	1.0008	0.3202	3.125	0.00384 **	-2.0478	1.4147	-1.448	0.15778
comp65967_c4_seq1 (Tbx20)	1.4641	0.3625	4.039	0.000448 ***	-1.0926	1.3812	-0.791	0.436333
comp66333_c0_seq1 (Scr)	0.9365	0.3169	2.955	0.00641 **	1.0401	1.5945	0.652	0.51974
comp66406_c1_seq1 (pnr)	0.6832	0.2215	3.084	0.00493 **	-1.6985	1.5594	-1.089	0.28644

Thoracic horn length

Target gene	Body size				dsRNA treatment			
	Estimate	Standard error	*t*	*P*	Estimate	Standard error	*t*	*P*
comp45679_c0_seq1 (Rx)	1.084	0.1791	6.053	2.15E-06 ***	-0.8611	0.9999	-0.861	3.97E-01
comp49439_c0_seq2 (Wnt7-1)	0.7693	0.1653	4.655	0.0000835 ***	-0.8975	1.0538	-0.852	0.402
comp61307_c0_seq1 (BarH1)	0.4646	0.1893	2.45E+00	2.02E-02 *	0.8606	0.8613	9.99E-01	0.3257
comp61421_c0_seq1 (Sox21b)	0.9463	0.1393	6.80E+00	3.27E-07 ***	-3.2362	0.7733	-4.19E+00	0.000288 ***
comp61925_c0_seq1 (dac)	0.6173	0.2115	2.919	0.00772 **	-4.564	1.3148	-3.471	0.00207 **
comp62820_c0_seq1 (Sox14)	0.334	0.1886	1.771	0.0905	9.1287	1.0749	8.49E+00	2.16E-08 ***
comp62938_c0_seq1 (Optix)	1.3729	0.3544	3.874	0.000464 ***	0.6698	1.5633	0.428	0.671038
comp63178_c0_seq2 (SP8)	0.6753	0.1798	3.76E+00	0.000839 ***	0.8474	1.1581	7.32E-01	4.71E-01
comp63338_c0_seq1 (otd2)	0.31	0.2521	1.23	0.227	1.4264	1.1011	1.295	0.204
comp63721_c0_seq1 (ab)	0.7974	0.1648	4.838	0.0000698 ***	11.8481	0.7769	15.25	1.62E-13 ***
comp65846_c0_seq1 (eyg)	0.8206	0.1685	4.87	0.0000312 ***	-0.1125	0.7444	-0.151	0.881
comp65967_c4_seq1 (Tbx20)	0.886	0.1965	4.509	0.000123 ***	-2.4353	0.8596	-2.833	0.008789 **
comp66333_c0_seq1 (Scr)	0.5816	0.2092	2.78	0.00978 **	8.0416	1.0527	7.639	3.24E-08 ***
comp66406_c1_seq1 (pnr)	0.1514	0.1147	1.32	0.199	28.6181	0.8076	35.436	<2e-16 ***
