# Supplementary material for: Rhinoceros beetle horn development reveals deep parallels with dung beetles
Source: PLoS Genet. 2018 Oct 4;14(10):e1007651. doi: 10.1371/journal.pgen.1007651 (PMC6171792; doi:10.1371/journal.pgen.1007651)
Supplement: S7 Table — (PDF) [file pgen.1007651.s015.pdf]

S7 Table. BDGP terms and function in appendages.

Transcript ID	Drosophila ortholog	Annotation symbol	Flybase id	BDGP term assigned		Function in appendages
				clypeolabrum	clypeo-labral primordia	
comp45679_c0_seq1	Retinal Homeobox	CG10052	FBgn0020617	Yes	Yes	
comp61307_c0_seq1	BarH1	CG5529	FBgn0011758			Yes
comp61421_c0_seq1	Sox21b	CG32139	FBgn0042630			
comp61925_c0_seq1	dacshund	CG4952	FBgn0005677			Yes
comp62820_c0_seq1	Sox box protein 14	CG3090	FBgn0005612			Yes
comp62938_c0_seq1	Optix	CG18455	FBgn0025360	Yes	Yes	
comp63178_c0_seq2	Sp1	CG1343	FBgn0020378	Yes	Yes	Yes
comp63721_c0_seq1	abrupt	CG43860	FBgn0264442			Yes
comp65967_c4_seq1	H15	CG6604	FBgn0016660			
comp65967_c4_seq1	midline	CG6634	FBgn0261963	Yes	Yes	
comp66333_c0_seq1	Sex combs reduced	CG1030	FBgn0003339			
comp66406_c1_seq1	pannier	CG3978	FBgn0003117			
