# Supplementary material for: Rhinoceros beetle horn development reveals deep parallels with dung beetles
Source: PLoS Genet. 2018 Oct 4;14(10):e1007651. doi: 10.1371/journal.pgen.1007651 (PMC6171792; doi:10.1371/journal.pgen.1007651)
Supplement: S8 Table — (PDF) [file pgen.1007651.s016.pdf]

S8 Table. Total RNA quality.

Samlple name	% DNA contamination	RNA integrity number
Male_1_headhorn	10.12	6.6
Male_1_thoracichorn	11.57	6.4
Male_2_headhorn	10.42	6.6
Male_2_thoracichorn	11.01	6.7
Male_3_headhorn	13.60	6.8
Male_3_thoracichorn	10.70	6.5
Female_1_headhorn	3.95	6.8
Female_1_thoracichorn	5.53	6.8
Female_2_headhorn	6.95	7.2
Female_2_thoracichorn	5.61	7.1
Female_3_headhorn	6.87	6.8
Female_3_thoracichorn	4.84	7.0
