# Supplementary material for: Rhinoceros beetle horn development reveals deep parallels with dung beetles
Source: PLoS Genet. 2018 Oct 4;14(10):e1007651. doi: 10.1371/journal.pgen.1007651 (PMC6171792; doi:10.1371/journal.pgen.1007651)
Supplement: S9 Table — (PDF) [file pgen.1007651.s017.pdf]

**S9 Table. Primers.**

*Trypoxylus dichotomus*

| ID | Gene | Forward (F) / Reverse (R) | Sequence (5´ to 3´) |
|---|---|---|---|
| comp3738_c0_seq1 | Wnt-10b | F | TTGGTCAGTATGTGGGACGA |
| | | R | AGCCGCTTCTCTAACATCCA |
| comp40568_c0_seq1 | Wnt6 protein precursor | F | AACCTGGTGGATTGTTCCTG |
| | | R | TGTTTCGCCATCTGGTATGA |
| comp45679_c0_seq1 | Retinal homeobox protein | F | AGTGAAACCGTTGAGGATGG |
| | | R | GAACTCTGACTTCGGGCAAG |
| comp47535_c0_seq1 | T-box protein Tbx1 >optomotor blind related gene 1 protein | F | CAATCAAATGGACGACAACG |
| | | R | ATTATGAGCGTCCTGCTGCT |
| comp49439_c0_seq2 | Wnt7-1 | F | GTTGGAACTGTACGGCGATT |
| | | R | GCACTTGCATTCAGTCAGGA |
| comp49933_c0_seq1 | bone morphogenetic protein 2-like | F | CCGGATTTGCATAAATTGCT |
| | | R | CCGTACGGAGCCAGAATAAA |
| comp52023_c0_seq1 | N, Calcium-binding EGF-like domain | F | TTACCGGTACAACGCGTACA |
| | | R | TACAGGCATCAGACCCATGA |
| comp52609_c0_seq1 | hormone receptor 83 | F | ATGGACTCGTATGACTTAGA |
| | | R | TTAAATTGTCACAACTACCC |
| comp56528_c0_seq1 | muscle LIM protein isoform 1 | F | TGCTTCAAGTGCAAGGATTG |
| | | R | GGCCGAAGAGTTTGCTGTAG |
| comp56805_c1_seq1 | cycle | F | TTGCTCCAAACAACAGCAAG |
| | | R | CCTAATCCAGCGTCTGCTTC |
| comp56808_c0_seq1 | smad nuclear interacting protein | F | TGATTTGATGGGGAAACCAT |
| | | R | CTCCTCGGCTTCTTTCCTTT |
| comp56914_c0_seq1 | brinker | F | CCGAAATCATCGTCCCTTTA |
| | | R | ATCGCTGCTTGATCTTTCGT |
| comp57995_c0_seq1 | Pox neuro | F | AAGGCCGTTACCAGATTGTG |
| | | R | TACTGTGCGTTGATGGCATT |
| comp58454_c0_seq1 | sloppy paired 2 | F | AGCCCCTTTAAAATCGTCGT |
| | | R | TTGCCAACCCTGCTTATTC |
| comp58528_c1_seq1 | odd-skipped | F | GGAGGATTTCAACACGGCTA |
| | | R | GCGTCCTTTCATGGATCAGT |
| comp59045_c0_seq1 | teashirt-like protein | F | AGCCTAGGCGCCTTAAGTTC |
| | | R | GATCCGGTATAACCCCGTCT |
| comp59576_c0_seq3 | Zinc finger, C3HC4 type (RING finger) | F | CAGTCGGATGGAACCTTTGT |
| | | R | AAACCCTTGAATTCCCCATC |
| comp59701_c0_seq2 | vestigial | F | GCGACCACAGAAAGAGGAAG |
| | | R | CCTGAGTACTGGGCCATGTT |
| comp59895_c0_seq1 | Antennapedia | F | CAGGACAACCTCCACAAGGT |
| | | R | GCTGTTGATGGTGTTGTTGG |
| comp60275_c0_seq1 | grain (dGATAc) | F | ATGCCTGTGGGCTGTATTTC |
| | | R | CCTTGTTCGTGTCCTGGATT |
| comp60275_c0_seq2 | grain (dGATAc) | F | ATGCCTGTGGGCTGTATTTC |
| | | R | CCTTGTTCGTGTCCTGGATT |
| comp61134_c0_seq1 | zinc finger MYM-type protein 1-like | F | ATGAGTGTTCGAGAAAAATT |
| | | R | CTACAAATTCCCAACCACTG |
| comp61360_c0_seq1 | TGF-beta family, myostatin>myoglianin | F | TCACGATGATGGTGTCAGGT |
| | | R | TTCGTCGAAGTACAGCATCG |
| comp61421_c0_seq1 | Sox21b | F | ATGTGCGACGGCGGTTTTAC |
| | | R | CTAGGTCGATATTTGTAATC |
| comp61853_c0_seq1 | epidermal growth factor-like protein | F | GCGGGTGCTTAAATGGTAAA |
| | | R | GGGCCTTCTTCCAATATCCT |
| comp61915_c0_seq1 | Lyra (senseless) | F | ATGAAAAAACATACCTACAT |
| | | R | TTAACTACTACTTGAGACCT |
| comp62113_c0_seq1 | Tnebrio hormone receptor 4 | F | ATCATCGTCATCATCATCAT |
| | | R | TTACAATAATAACAATAAAT |
| comp62820_c0_seq1 | SOX-14 | F | CATGGTGTGGTCGCAAATAG |
| | | R | CGTTGGCGTATTTGTTGATG |
| comp62938_c0_seq1 | Optix | F | ACGAAAAGCTCTCACGGAAA |
| | | R | ACTCGCTAATTGGGCTGCTA |
| comp63178_c0_seq1 | Sp-like zinc finger transcription factor (Sp8) | F | TGCTCTTCGCCTTTTCGCTA |
| | | R | CGCGTTCGCAAAGACGTTAT |
| comp63338_c0_seq1 | orthodenticle-2 | F | CGGCATGGCTTACTTGAAAT |
| | | R | CTGGCGGTGAAGATTTTGTT |
| comp63540_c0_seq1 | epidermal growth factor-like protein | F | CCAAGCGGAAGATATTGCAT |
| | | R | AGAGCGCAGAAGGGAATACA |
| comp63721_c0_seq1 | abrupt | F | CCGTTACCCCAAAGACTGAA |
| | | R | AGATTTCGCAACGGTACCAC |
| comp63774_c2_seq1 | disco-related | F | AACGGCCAATTTATCAGCAC |
| | | R | AGTTCGATTCCAGGATGTGG |
| comp64312_c0_seq1 | BMP and activin membrane-bound inhibitor-like | F | GCAATTCCGAAGTGTGGTTT |
| | | R | GCGTGTATTTCGCTGACGTA |
| comp64379_c1_seq3 | engrailed | F | TTCTTGATCTTCGCCCGCTT |
| | | R | GCGTTCGAGGATAGGTCCAG |
| comp65258_c1_seq1 | drumstick | F | ATGTTTGCGATAATGCAACT |
| | | R | TTATCGCCATATGCACTGGC |
| comp65763_c0_seq1 | iroquois-class homeodomain protein | F | CCGATAACGGGACAGAGTGT |
| | | R | TGGTCGGATACGGATTCTTC |
| comp65832_c1_seq1 | Winged helix-turn helix, Homeodomain-like domain | F | ATGTTGATGAAAGATTTGAA |
| | | R | TTATGACTTCTGGAAATGCA |
| comp65846_c0_seq1 | eyegone | F | AGCCGAAATTCCGAAGAAAT |
| | | R | TGCCTTGAAAGCGCTATTTT |
| comp65931_c0_seq1 | optomotor-blind-like | F | TCCCCGTCTAGTACGTCACC |
| | | R | GTCTGCAGCTACGATGTCCA |
| comp65967_c4_seq1 | T-box transcription factor 20 | F | CCTTCGAAAAGGTCAAGCTG |
| | | R | ACAGTTGAGACCTGGCTGCT |
| comp66046_c4_seq1 | spalt | F | TGCATCAGTGTCCCGTATGT |
| | | R | GTTTACGTTCAGGCCGATGT |

| | | | |
|---|---|---|---|
| comp66333_c0_seq1 | Sex combs reduced | F | GTGCGAGCAACGTTACGATA |
| | | R | CGTCAGGTACCGGTTGAAGT |
| comp66406_c1_seq1 | pannier | F | CGTCAATTGTGGGGCTATCT |
| | | R | CCACTGACGAGTTCCCATTT |
| comp102925_c0_seq | brachyenteron (Brachyury (T) homolog) | F | CGCTCAACAGCAAACACAAT |
| | | R | TTGATGGCCGGACTTAAAAC |
| comp786117_c0_seq | Transcription factor 21 (Podocyte-expressed 1) (Pod-1) (Epicardin) (Capsulin) | F | CAAGCGACGCATAACAGAAT |
| | | R | TCATTCCAAGACAAATCACGA |

*Tribolium castaneum*

| Gene | Forward (F) / Reverse (R) | Sequence (5´ to 3´) |
|---|---|---|
| Retinal homeobox protein | F | TGAGTACCACGCCGTCTCCT |
| | R | CTTGGCTTTGAGACGCAGCG |
| Optix | F | GGCCGCCGCTAAGAATAGGA |
| | R | AGAATGGGGCCCGAAACGAA |
| Tbx20 | F | CAGTTGTTTCTGAGGGCGGC |
| | R | TCAAATGCGAACTTCCGGCG |
| SP8 | F | ATGCTTACTGATATGACCCC |
| | R | CACGGATGGAAGCCCTTGCC |
| BarH1 | F | CGGACCATGCCTCAAACTGC |
| | R | TCCCGTCTGACCGTTGGAAC |
| dac | F | AGGAATGCCCACGATGGAGG |
| | R | GAATGGTCGCCGTCCCCTAA |
| Sox14 | F | TGATGACGCCCCTTCCACTG |
| | R | ATCTCGATGTCCGCCAGCTC |
| abrupt | F | ACAACGACCCCGAAGACCTG |
| | R | GCCTGCTCCGAGGACATCAT |

Control

| Gene | Forward (F) / Reverse (R) | Sequence (5´ to 3´) |
|---|---|---|
| egfp | F | AGACCACATCATGGCCGACAAGCAGAA |
| | R | AGACCACAACTCCAGCAGGACCATGTG |

* All primers have T7 sequence (taatacgactcactataggg) at 5´ end.
