# Supplementary material for: Rhinoceros beetle horn development reveals deep parallels with dung beetles
Source: PLoS Genet. 2018 Oct 4;14(10):e1007651. doi: 10.1371/journal.pgen.1007651 (PMC6171792; doi:10.1371/journal.pgen.1007651)
Supplement: S7 Appendix — (DOCX) [file pgen.1007651.s024.docx]

**S7 Appendix. Summary of alternative data analyses.**

Since some new methods for sequence data analysis have appeared after our initial data analysis, we here show the results of data reanalysis using some alternative methods.

Trinity de novo assembly with the latest version, and with the trimmomatic option

| No. | Trinity version  (release date) | Trimmomatic option | Total trinity genes | Total trinity transcripts | All transcript contigs | Only longest isoform per gene |
| --- | --- | --- | --- | --- | --- | --- |
| 1 | r2012-06-08  (8-Jun-2012) | No | 82,108 | 127,986 | 3,158 | 1,617 |
| 2 | v2.6.5  (14-Feb-2018) | No | 87,622 | 164,170 | 2,629 | 1,636 |
| 3 | v2.6.5  (14-Feb-2018) | Yes | 85,178 | 157,476 | 2,803 | 1,716 |

The number of both total trinity genes and transcripts increase with the newer improved version of Trinity software. It may be valuable to repeat quantification and DEG analysis with such transcriptomes with increased number of transcripts for finding unidentified horn formation genes.

Spearman’s rank correlation between p-values calculated from two different analysis pipelines

Male head horn vs Female head horn

| Analysis1 | | Analysis2 | | Spearman’s rank correlation |
| --- | --- | --- | --- | --- |
| RSEM | edgeR | Kallisto | edgeR | 0.6013768 |
| RSEM | edgeR | Salmon | edgeR | 0.5936978 |
| kallisto | edgeR | Salmon | edgeR | 0.9498206 |

Male thoracic horn vs Female thoracic horn

| Analysis1 | | Analysis2 | | Spearman’s rank correlation |
| --- | --- | --- | --- | --- |
| RSEM | edgeR | Kallisto | edgeR | 0.562333 |
| RSEM | edgeR | Salmon | edgeR | 0.5511436 |
| kallisto | edgeR | Salmon | edgeR | 0.9488589 |

Male head horn vs Male thoracic horn

| Analysis1 | | Analysis2 | | Spearman’s rank correlation |
| --- | --- | --- | --- | --- |
| RSEM | edgeR | Kallisto | edgeR | 0.31639 |
| RSEM | edgeR | Salmon | edgeR | 0.308661 |
| kallisto | edgeR | Salmon | edgeR | 0.9201739 |

Female head horn vs Female thoracic horn

| Analysis1 | | Analysis2 | | Spearman’s rank correlation |
| --- | --- | --- | --- | --- |
| RSEM | edgeR | Kallisto | edgeR | 0.7076461 |
| RSEM | edgeR | Salmon | edgeR | 0.6925607 |
| kallisto | edgeR | Salmon | edgeR | 0.9483829 |

Comparison of the number of differentially expressed genes (FDR < 0.05) among different pipelines

RSEM > edgeR

|  | Group 1 | Group 2 | High in Group1 | High in Group2 | Total |
| --- | --- | --- | --- | --- | --- |
| Intersexual | Male HH | Female HH | 123 | 440 | 563 |
|  | Male TH | Female TH | 99 | 77 | 176 |
| Intrasexual | Male HH | Male TH | 77 | 301 | 378 |
|  | Female HH | Female TH | 321 | 115 | 436 |

Salmon > tximport > edgeR

|  | Group 1 | Group 2 | High in Group1 | High in Group2 | Total |
| --- | --- | --- | --- | --- | --- |
| Intersexual | Male HH | Female HH | 185 | 69 | 254 |
|  | Male TH | Female TH | 96 | 81 | 177 |
| Intrasexual | Male HH | Male TH | 0 | 0 | 0 |
|  | Female HH | Female TH | 245 | 101 | 346 |

kallisto > tximport > edgeR

|  | Group 1 | Group 2 | High in Group1 | High in Group2 | Total |
| --- | --- | --- | --- | --- | --- |
| Intersexual | Male HH | Female HH | 63 | 168 | 231 |
|  | Male TH | Female TH | 75 | 94 | 169 |
| Intrasexual | Male HH | Male TH | 0 | 0 | 0 |
|  | Female HH | Female TH | 243 | 100 | 343 |

We quantified RNA-seq reads with two alignment-free methods, Kallisto and Salmon, and identified DEGs with edgeR. Tximport was used for summarizing transcript level data to gene level. We compared the number of DEGs at false discovery rate less than 0.05 from three different pipelines, and found that alignment-free quantifications result in highly different outputs as spearman’s rank correlation scores between p-values after different pipelines indicates about 0.3 to 0.7. The correlation of p-values between two alignment-free methods is as high as over 0.92.
